# Dynamic alterations in linear growth and endocrine parameters in children with obesity and height reference values

**DOI:** 10.1016/j.eclinm.2021.100977

**Published:** 2021-06-23

**Authors:** Elena Kempf, Mandy Vogel, Tim Vogel, Jürgen Kratzsch, Kathrin Landgraf, Andreas Kühnapfel, Ruth Gausche, Daniel Gräfe, Elena Sergeyev, Roland Pfäffle, Wieland Kiess, Juraj Stanik, Antje Körner

**Affiliations:** a University of Leipzig, Medical Faculty, University Hospital for Children and Adolescents, Center for Pediatric Research, Liebigstr.19, Leipzig 04103, Germany; b University of Leipzig, Medical Faculty, LIFE–Leipzig Research Center for Civilization Diseases, Philipp-Rosenthal-Str. 27, Leipzig 04103, Germany; c University of Leipzig, Medical Faculty, Institute of Laboratory Medicine, Clinical Chemistry and Molecular Diagnostics, Liebigstr. 27b, Leipzig 04103, Germany; d University of Leipzig, Medical Faculty, Institute for Medical Informatics, Statistics and Epidemiology (IMISE), Härtelstr. 16-18, Leipzig 04107, Germany; e University of Leipzig, Medical Faculty, Growth Network CrescNet, Liebigstr. 20a, Leipzig 04103, Germany,; f University of Leipzig, Medical Faculty, Pediatric Radiology, Liebigstr. 20a, Leipzig 04103, Germany; g Comenius University, Medical Faculty and Children Faculty Hospital, First Department of Pediatrics, Limbova 1, 833 40 Bratislava, Slovakia and Slovak Academy of Sciences, Biomedical Research Center, Institute of Experimental Endocrinology, DIABGENE Laboratory, Dúbravská cesta 9, Bratislava 845 05, Slovakia

**Keywords:** Children, Obesity, Height, Growth velocity, IGF-1, Insulin, Leptin, Estradiol, Testosterone

## Abstract

**Background:**

Obesity can affect linear growth of children but there is uncertainty regarding the dynamics and potential causes.

**Methods:**

In the population-based LIFE Child and the obesity-enriched Leipzig Obesity Childhood cohorts (8,629 children, 37,493 measurements), recruited from 1999 to 2018 in Germany, we compared height, growth, and endocrine parameters between normal-weight and children with obesity (0–20 years). Derived from the independent German CrescNet registry (12,703 children) we generated height reference values specific for children with obesity (data collected from 1999 to 2020).

**Findings:**

Children with obesity were significantly taller than normal-weight peers, differing at maximum by 7·6 cm (1·4 height, standard deviation scores or SDS) at age 6–8 years. Already at birth, children with obesity were slightly taller and thereafter had increased growth velocities by up to 1·2 cm/year. This growth acceleration was unrelated to parental height, but was accompanied by increased levels of insulin-like growth factor-1 (IGF-1), insulin and leptin. During puberty, children with obesity showed a catch-down in height SDS. The reduction in pubertal growth velocity by up to 25% coincided with a decrease in levels of IGF-1 (by 17%) and testosterone (by 62%) in boys and estradiol (by 37%) in girls. We confirmed these alterations in growth in the independent CrescNet cohort and furthermore provide height reference values for children with obesity for open access.

**Interpretation:**

Dynamics of linear growth are altered distinctively in different developmental phases in children with obesity. Early emergence before other profound comorbidities implies predisposition, environmental, and/or endocrine factors affecting growth in early life. Height reference values for children with obesity may enhance the precision of clinical health surveillance.

**Funding:**

German Research Foundation, German Diabetes Association, EU, ESF, ERDF, State of Saxony, ESPE, Hexal, Novo Nordisk, Pfizer Pharma.

**Research in context**

**Evidence before this study**

We searched PubMed for articles published up to 1 April 2021 using the terms: growth/ height /IGF-1/ estrogen/ estradiol/ testosterone AND obese/ obesity AND children/ adolescents. Alterations in growth of children with obesity were reported, but distinct and controversial in direction across the age span and between studies. Hence, whether, which and when differences in growth dynamics manifest and potential reasons are not clear.

**Added value of this study**

This comprehensive study characterizes significant deviations in linear growth and endocrine parameters in boys and girls with obesity across the entire age span from birth to young adulthood compared to normal-weight peers. Until adolescence, children with obesity are taller independent of familial predisposition, which is attributable to increased birth size with subsequent growth acceleration until puberty with concomitant elevation of IGF-1 and metabolic alterations such as hyperinsulinemia. The blunted pubertal growth spurt in children with obesity with concurrent alterations in sex hormone profiles eventually results in convergence of height curves in adolescence. We provide height reference values specifically for children with obesity.

**Implications of all the available evidence**

Our findings add deviations in growth and endocrine factors to the array of obesity-related complications in children. The early emergence implies that predisposition, environmental, and/or endocrine factors affect growth in early life. The freely accessible height reference values specific for children with obesity may enhance the precision of clinical health surveillance.

Alt-text: Unlabelled box

## 1 Introduction

Childhood obesity is not only a risk factor for serious cardio-metabolic comorbidities with sustained consequences in increasing morbidity and mortality in adult life [1]. It has also been hypothesized that obesity during childhood affects growth [2,3]. Alterations in linear growth were reported to be associated with more adverse adult cardiovascular risk profile implying that advanced early linear growth is related to negative cardiovascular health outcomes such as coronary heart disease, even independently from body mass index (BMI) [4,5]. Furthermore, more detailed insight into the dynamic association between body weight and growth may contribute to the understanding of the complex relationships of energy balance, hormonal milieu, and growth in the development of obesity.

The growth of children with obesity was reported to be enhanced [2,3] or blunted [6]. Those differences in observations might derive from the distinct age ranges and developmental stages of the children investigated in those studies. Previous studies showed that children with obesity were taller between ages 1–14 years [2] and that BMI gain at ages 2–8 years was associated with increased height gain in early childhood and reduced height gain in adolescence, but not with final height [3,6]. In contrast, it was also reported that in relation to parental height, final height of individuals with obesity was compromised [7]. However, there is no comprehensive study spanning the entire age range from childhood to adolescence addressing whether, which and when exactly alterations in growth dynamics in children with obesity occur and that may point to potential endocrine mechanisms underlying the altered growth in obesity.

For clinical management and individual health surveillance, it is important to correctly assess if a child's growth deviates from the expected pattern and may hence be suggestive for other pathologies, e.g. underlying syndromic or monogenetic disorders [8]. In this regard, it is also of particular importance to recognize less evident and more subtle growth alterations. With evidence for growth alterations in the population of children with obesity, height reference values for children with obesity might help to avoid misinterpretation and unnecessary medical diagnostic work-up for tall stature, growth acceleration or precocious puberty in children with obesity. Also, growth alterations of children with obesity being too small for their age may be overlooked when applying the current reference values. Therefore, height reference values specifically for children with obesity are needed to allow clinicians an appropriate assessment of a child's height for its weight status.

Several hormones are involved in regulating growth, and their levels may be altered with obesity [9,10]. The growth hormone (GH)/insulin-like growth factor-1 (IGF-1) axis is the major endocrine system regulating growth [9,11,12]. GH serum levels in children with obesity tend to be reduced [11], while the association of obesity with IGF-1 is inconsistent and seems to be related to age [12], [13], [14]. Production, bioavailability, and signaling of IGF-1 are modulated by IGF-binding proteins (IGFBPs) [9], thyroid hormones [15], and sex steroids [16]. Particularly, insulin as an anabolic hormone might affect growth by suppressing GH secretion [9], or, due to structural similarity to IGF-1, by interfering with IGF-1 signaling and IGFBPs levels [9]. Another hormone potentially accelerating growth is adipose tissue-derived leptin [9], with effects on the GH axis, pubertal development [10] and bone formation [17]. Since growth as well as most of those hormones are dynamically regulated during development, serum levels must be tracked throughout entire childhood in order to detect associations with obesity-related differences in growth.

We hypothesized that height of children with obesity is altered compared to normal-weight children. We were particularly interested in the temporal dynamics across age. We further addressed how potential influencing factors such as parental height, size at birth or changes in hormonal and metabolic factors are related to the dynamic alterations in growth. We finally generated reference values for height specifically for children with obesity in order to provide a solid basis for appropriate height assessment in children with obesity.

## 2 Methods

### 2.1 Study populations for comparative assessments

Our study encompassed 8,629 children with 37,493 observations across ages 0–20 years from the population-based LIFE Child [18,19] and the obesity-enriched Leipzig Obesity Childhood Cohort [20] (detailed description provided in Additional file 1, Ia).

Main outcome parameters (BMI, height, growth velocity) were referenced to sex and age according to current guidelines [21,22] and are given as standard deviation scores (SDS).

Based on assessment of fit in normal distribution plots (Additional file 1, Ib), we included boys and girls with height SDS between −2·5 and 4·0 and BMI SDS≥−3·5. Children suffering from diseases such as type 1 diabetes, syndromes and other conditions with severe disability (permanent immobility, cerebral palsy etc.) or with manifest precocious puberty, intracranial hypertension or valproate medication were excluded, as this could affect growth and development. Likewise, we excluded individuals taking medications affecting growth (e.g. growth hormone, systemic glucocorticoids, immunosuppressives). Exclusively for analyzing insulin and Homeostatic Model Assessment-Insulin Resistance (HOMA-IR) or thyroid hormones, data of children taking metformin or thyroxin, respectively, were excluded (Additional file 1, Ic).

Observations of children born prematurely (gestational week<38) were included from age at measurement ≥2·0 years onwards as by then they are supposed to have caught up in height [23]. As such, by then height SDS did not differ significantly anymore between pre-term and full-term normal-weight children (not shown). An overview of the characteristics of the study sample stratified by weight categories and age groups (one observation per individual per age group) is shown in Additional files 1, Id, and Ie, respectively.

Including all observations, children had a minimum of 1 observation and maximum of 17 observations. 4,113 children (47·7%) had at least one follow-up visit (Additional file 1, If).

Written consent was obtained from parents and assent from children ≥12 years. The study was approved by the ethics committee of the University of Leipzig and is registered in the clinical trials data base (NCT02550236, NCT04491344).

### 2.2 Independent study population for generation of height reference values

Height reference values for children with obesity were generated from data of 12,703 subjects derived from the German CrescNet patient registry [2,24]. Within this network, pediatricians regularly report pseudonymized weight and growth data from children seen for well-child visits or other consultations. The registry was approved by the Federal Saxonian Data Protection Authority and is registered at the clinical trials database (NCT03072537).

Detailed descriptions of the CrescNet registry are provided in Additional file 1, IIa. Inclusion criteria for the generation of height reference values were defined according to the criteria of the study above and provided in Additional file 1, IIb.

### 2.3 Anthropometric measurements

Anthropometric measurements were performed using standardized procedures by regularly trained and certified health care professional staff. Height was measured to the nearest millimeter using a rigid stadiometer. Weight was measured in light underwear to the nearest 0·1 kg using a calibrated scale. Growth velocities for children at age ≥1·5 years (mean age between the two height observations) were calculated using consecutive follow-up data (minimum interval length of 3 months, maximum 2 years). Growth velocities <0 cm/year were excluded as well as of <1 cm/year for children aged <14 years as they were inconclusive since children are supposed to grow at least 1 cm/year until the age of 14 years [22]. Growth velocity SDS values >10 were excluded as they were implausibly high and may result from measurement errors or undocumented disease. Assignment into the weight categories was based on the weight status at the first anthropometric observation.

Pubertal stage was assessed according to Tanner criteria as described in Additional file 1, III, Table S4. Birth length and weight were recorded and SDS were calculated according to Voigt and corrected for gestational age [25]. Further explanation for birth and parental parameters are provided in Additional file 1, IV.

### 2.4 Laboratory measurements

Serum parameters were measured by standard laboratory procedures in the Institute of Laboratory Medicine, Clinical Chemistry and Molecular Diagnostics of the University of Leipzig, Germany, by the following methods: IGF-1 and IGF-binding protein 3 (IGFBP-3) were measured by the automated immunoluminometric assay (Advantage, Nichols Institute Diagnostics, San Clemente, CA, USA) [26], the enzyme-linked immunosorbent assay (ELISA) (Mediagnost, Reutlingen, Germany), and the automated chemiluminescence immunoassay (CLIA) iSYS (IDS, Boldon/Tyne & Wear, UK) [27,28]. We obtained qualitatively comparable results if each method was analysed separately. Leptin was measured using an ELISA (Mediagnost, Reutlingen, Germany), glucose by photometric measurement (Cobas Roche, Mannheim, Germany) and insulin by CLIA (Liaison, DiaSorin, Dietzenbach, Germany) or by electroCLIA (ECLIA) (Cobas Roche, Mannheim, Germany), following an overnight fast. Estradiol and testosterone were measured using ECLIA by Elecsys 2010 or by Cobas fully automated immunoassay systems (Cobas Roche, Mannheim, Germany). Sex hormone-binding globulin (SHBG), luteinizing hormone (LH), follicle-stimulating hormone (FSH), dehydroepiandrosterone sulfate (DHEA-S), thyroid-stimulating hormone (TSH), and free thyroxin (FT4) were measured using ECLIA (Cobas Roche, Basel, Switzerland). Homeostatic Model Assessment for Insulin Resistance (HOMA-IR) was calculated as HOMA-IR=(Insulin[mU/l] × Glucose[*mmol*/*l*])/22.5 [29].

### 2.5 Statistical analysis

Data preparation and statistical analyses were performed using Statistica 10 (Dell, Round Rock, US), GraphPad Prism 6 (GraphPad Software, San Diego, CA) and R (Version 3.5.0, Vienna, Austria). *P***-**values <0·05 were considered statistically significant.

#### 2.5.1 Comparison of children of different weight categories

Children were allocated to age groups according to their rounded age (e.g. age group 1: ≥0·5 and <1·5 years; age group 2 ≥ 1·5 and <2·5 years etc.). Children older than 17·5 years were allocated to the group 18+. To avoid bias by several observations of an individual within one age group, only the first observation of an individual per age group was included. For each visit children were classified into one of the following weight categories according to the current BMI SDS as recommended by the German Working Group for Pediatric Obesity Consensus Guideline [30]: underweight (BMI SDS <−1·28), normal-weight (−1·28≤ BMI SDS ≤1·28), overweight (1·28< BMI SDS ≤1·88) or with obesity (BMI SDS >1·88). For visualization of growth velocities the children were allocated to age groups accordingly by using the rounded mean age between the two height observations. Only the first growth velocity of an individual per age group was included.

For visualization of the differences in height, growth, and endocrine factors between normal-weight and children with obesity, data of the normal-weight and subgroups with obesity were presented by age group as mean±standard error (SEM) in a cross-sectional manner. The mean values between normal-weight and children with obesity per age group were compared using unpaired t-tests with Holm-Šídák correction for multiple comparisons. We excluded age-weight-group strata from charts if sample size (N) was <6 per sex/age group/weight category.

#### 2.5.2 Generalized additive models

Using the data set with multiple observations per individual per age group including 37,493 measurements from 8,629 children (Additional file 1, Ic, Figure S2), we modeled the age trend for height SDS, IGF-1, and height velocity non-parametrically by applying generalized additive models considering multiple measurements per subject by adding a random effect for the subjects assuming a simple Gaussian random effect.

#### 2.5.3 Effect of BMI SDS on height SDS in the following year

We estimated the effect of BMI SDS of children of all weight classes (at time point t) on their height SDS in the following year (time point *t* + 1 year) and adjusted for current height SDS (at time point t). Data are presented as effect estimates with standard errors across the age groups (at time point t) in a one-year resolution and are marked with an asterisk if the association was significant. Here also the data set including multiple observations per individual per age group (Additional file 1, Ic, Figure S2) was used.

#### 2.5.4 Generalized additive mixed models and multivariate regression analyses

For evaluation whether mid-parental height and birth parameters contributed to the observed age-dependent increase in height SDS in prepubertal children with obesity, we applied generalized additive mixed models in order to model the trend of height SDS dependent on age in normal-weight and in children with obesity. The data set including multiple observations per individual per age group (Additional file 1, Ic, Figure S2) was used. To account for multiple measurements per subject, random effects were included on the intercept. Subsequently, cut points were identified by visual inspection to receive three age intervals of an approximately linear trend, for boys 2·0–4·99, 5·0–10·99 and 11·0–17·0 years and for girls 2·0–8·249, 8·25–10·99 and 11·0–17·0 years. For each age interval, hierarchical linear regression analysis was applied: First, regression analyses with only age as independent and height SDS and weight group as dependent variables have been performed. Second, hierarchical linear multivariate regression analysis has been performed with height SDS as dependent variable and age, mid-parental height SDS and birth length and weight SDS and their two-way interactions as independent variables. Step-wise deletion revealed that birth weight SDS as well as the two way interaction between the other covariates did not add any information beyond the information already contained in the model and were therefore removed from the model. Both analyses have also been accounted for multiple measurements as described above.

#### 2.5.5 Generation of reference values for height for children with obesity

For the generation of height reference values for German girls and boys with obesity, one randomly selected observation of each child with obesity from the CrescNet registry was used. Reference values were estimated for ages of 0 to 18 years using the LMS-method [31] and using the same modeling parameter as Kromeyer-Hauschild et al. 2001 [21]. The novel height reference values for children with obesity are applicable at the CrescNet website [32] and, furthermore, were implemented into the Ped(Z) app that can be accessed publically at the Ped(Z) Pediatric Calculator website [33]. In addition, an R package is available [34].

### 2.6 Role of the funding source

The funders of the study had no role in study design, in collection, analysis, and interpretation of data or writing of the report. The authors had full access to the full data in the study and accept responsibility to submit for publication.

## 3 Results

### 3.1 Children with obesity are taller than normal-weight peers during early childhood

**Fig. 1:**
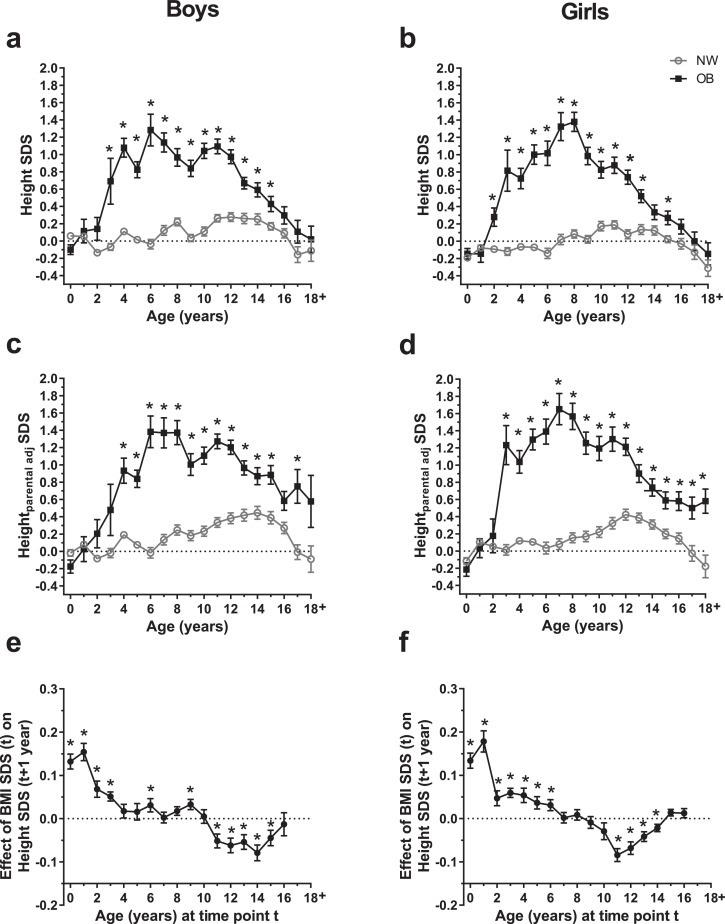
**Growth patterns of children with normal-weight and obesity and effect of BMI on subsequent height.** Parameters for height are presented across age groups 0–18+ years for boys and for girls separately. a, b: Height standard deviation score (SDS), c, d: Height SDS relative to mid-parental height SDS (Height_parental adj_ SDS). For a-d data are stratified for obesity (OB, full black squares) and normal-weight (NW, open gray circles) and are shown as mean with standard error; Asterisks (*) mark significant (*p*<0·05) differences between NW and OB groups assessed by multiple t-tests combined with a Holm-Šídák multiple comparison test. Sample sizes are provided in Additional file 1, Ie. e, f: Effects of body mass index (BMI) SDS (at time point t) on height SDS in the following year of age (at time point *t* + 1 year) are presented; data are shown as effect estimates with standard errors and are marked with asterisks (*) if an association was significant (*p*<0·05).

Children with obesity were significantly taller than their normal-weight peers. While the normal-weight group showed a height SDS around the expected 0 SDS throughout childhood, the group with obesity had an elevated height SDS around 1 SDS indicating relative tall stature (Fig. 1*a*+*b*). The most pronounced difference appeared around the age of 6 years in boys and 8 years in girls, accounting for a total difference in height of 6·8 and 7·6 cm, respectively (Additional File 1, VII, Figure S10 *a*+*b*). Beyond the age of 12 years in boys and 9 years in girls, the height SDS curves of the children with obesity and lean group converged, and there were no significant differences in final height. Also, we repeated the analyses with exclusion of all data from prematurely-born children and obtained the same results (not shown).

Furthermore, we modelled height dependent on age by including multiple measurements and, at the same time, reducing potential bias due to overrepresentation of subjects with repetitive measurements (Additional file 1, V, Figure S7a). This approach showed essentially the same pattern of increased height in the obesity group during childhood and a catch-down thereafter as shown before in Fig. 1*a*+*b*.

### 3.2 Growth acceleration in children with obesity is independent from parental height and birth length

As familial predisposition is a major determinant for height, we corrected for mid-parental height (Height_parental adj_ SDS) and found a similar pattern indicating that children with obesity were taller independent of familial predisposition (Fig. 1*c*+*d*). However_,_ when corrected for parental height, individuals with obesity had an increased final height compared to normal-weight peers. We did notice, however, that the parents of children with obesity in our study were slightly shorter than parents of normal-weight children (Additional file 1, IVa).

Next, we investigated the influence of birth weight and length on later heights, as we observed that, independent from weight status, height SDS of 4–14 years old children were lower in previously small (SGA) than in appropriate (AGA) and particularly large for gestational age (LGA) born children (Additional file 1, IVb, Figure S5).

Particularly girls with obesity had already been heavier at birth by >110 g and also birth length was higher compared to their normal-weight peers (Additional file 1, IVb, Table S6).

We investigated more in detail if mid-parental height or birth length were associated with this prepubertal increase in height SDS observed in children with obesity (Fig. 1*a*+*b*). To address this question, we modeled the differences in height SDS across age between normal-weight and children with obesity (Additional file 1, IVc, Figure S6) and defined three age intervals according to the slopes. In the first age interval, in boys as well as in girls, regression analyses showed significant differences in age trends between the weight groups with a steeper slope of height SDS in the obesity group compared to the normal-weight group (Additional file 1, IVc, Table S7). Between ages of 5–11 years in boys and 8·25–11 years in girls, height SDS plateaued and decreased beyond the age of 11 years, with a steeper slope observed in children with obesity.

We found that in boys as well as in girls, mid-parental height SDS and birth length SDS were significant predictors of height SDS in lean as well as in children with obesity in all age intervals (Additional file 1, IVc, Table S7). However, inclusion of the parameters mid-parental height SDS and birth length SDS into the model did not alter the association of age with height SDS in children with obesity or explain the difference in trends between the two groups. This suggests that mid-parental height and birth length determine absolute height, but are not associated with the obesity-related differences in growth dynamics.

### 3.3 BMI SDS is prospectively associated with height SDS

In order to examine the temporality of weight status and subsequent height of the children, we analyzed the effect of current BMI SDS on height SDS in the following year adjusted to the current height SDS. In early childhood associations between BMI SDS and subsequent height SDS were positive until the age of 10 and 8 years in boys and girls, respectively (Fig. 1*e*+*f*). The strongest effect occurred at the age of 1 year, where a BMI elevation of 1 SDS implied an average increase of around +0·18 units of height SDS in the following year. After the age of 11 years, BMI SDS had negative effects on height SDS in the following year with a negative peak at the age of 14 years in boys and 11 years in girls. Vice versa, tendencies were similar for the effect of height SDS on BMI SDS in the following age, adjusted for BMI SDS at the current age.

### 3.4 Increased growth velocity in early childhood and blunted pubertal growth spurt in children with obesity

**Fig. 2:**
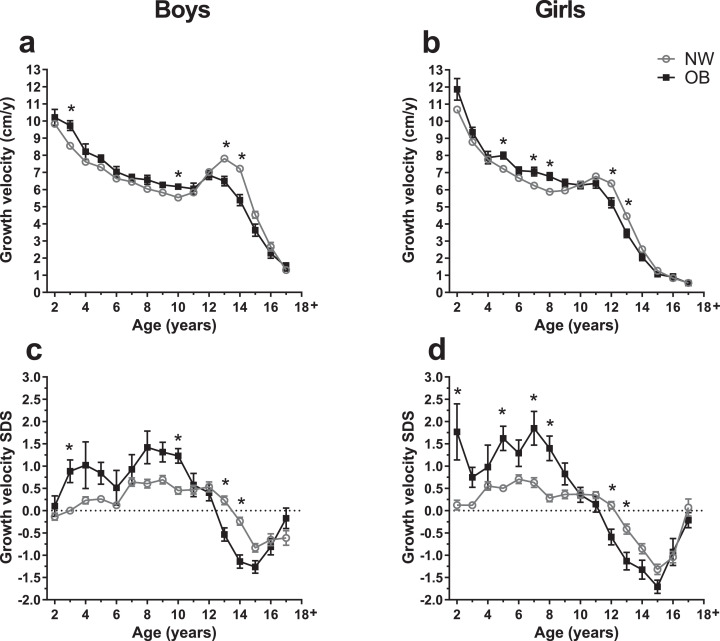
**Growth velocities of children with normal-weight and obesity.** Growth velocities are depicted for with obesity (OB, full black squares) and normal-weight (NW, open gray circles) groups of boys and girls for age groups 2–18+ years. Sample sizes are provided in Additional file 1, Ie. a, b: Total growth velocities in cm per year (cm/y); c, d: Growth velocity standard deviation scores (SDS); Data are shown as mean with standard error; Asterisks (*) mark significant (*p*<0·05) differences between NW and OB groups assessed by multiple t-tests combined with a Holm-Šídák multiple comparison test.

In a subcohort of children with growth velocities available, we assessed whether the obesity-related increase in height in early childhood was due to accelerated growth velocities. The height SDS patterns of this subgroup were not different from the entire cohort (not shown). Indeed, growth velocity (SDS) was slightly higher at ages 2–11 for boys with obesity and at ages 2–10 for girls with obesity compared to normal-weight children, with an increase of approximately 1 cm/year (Fig. 2).

In the normal-weight group, the increase in growth velocity during pubertal growth spurt was evident at the age of 13 years in boys and 11 years in girls as expected. However, in children with obesity, such a prominent growth spurt was lacking, further reflected by significantly lower growth velocities with reductions by up to 25% in boys aged 13–14 and by 22% in girls aged 12–13 years compared with their normal-weight peers.

When modeling the growth velocities, we received comparable results (Additional file 1, V, Figure S7b).

### 3.5 Alterations in pubertal timing and endocrine parameters in children with obesity

Boys with obesity were on average 6·6 months older than normal-weight boys when entering puberty (Additional file 1, III, Table S5). In contrast, girls with obesity were 9·1 months younger and experienced menarche 8·9 months earlier than normal-weight girls.

Serum levels of IGF-1 or IGFBP-3, sex hormones, metabolic factors, and thyroid hormones were available in subcohorts (Additional file 1, Ie). Growth velocities of the subcohorts are shown in the graphs and did not differ from those of the entire cohort.

**Fig. 3:**
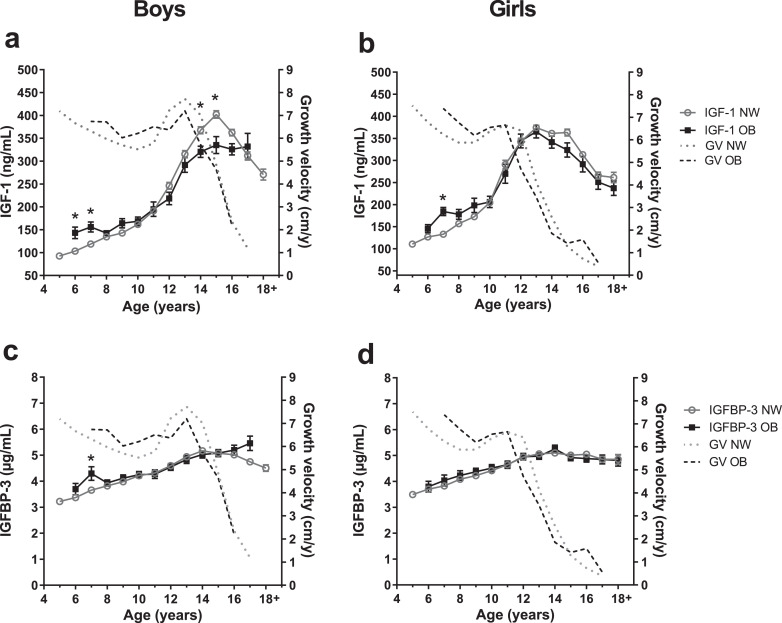
**Serum IGF-1 and IGFPB-3 levels of children with normal-weight and obesity.** Serum hormone levels of with obesity (OB, full black squares) and normal-weight (NW, open gray circles) groups of boys and girls are presented for age groups 5–18+ years. Data are shown as mean with standard error. The gray dotted-lined curves presents the growth velocities (GV) in cm per year (cm/y) of normal-weight individuals from whom insulin-like growth factor-1 (IGF-1) or IGF-binding protein 3 (IGFBP-3) measurements were available. The black dashed-lined curves show growth velocities from individuals with obesity from whom IGF-1 or IGFBP-3 measurements were available. Growth velocities are presented as mean without standard error. Sample sizes for this IGF-1 or IGFBP3 subcohort are provided in Additional file 1, Ie. a, b: Serum IGF-1 in ng/mL; c, d: Serum IGFBP-3 in µg/mL. Asterisks (*) mark significant (*p*<0·05) differences between NW and OB groups assessed by multiple t-tests combined with a Holm-Šídák multiple comparison test.

IGF-1 levels were elevated in children with obesity at ages 6–9 years, corresponding to the elevated growth velocities (Fig. 3*a*+*b* and modeled in Additional file 1, V, Figure S7c). In contrast, during pubertal age IGF-1 levels decreased by up to 17%. There were no major differences in IGFBP-3 levels between the weight groups (Fig. 3*c*+*d*).

**Fig. 4:**
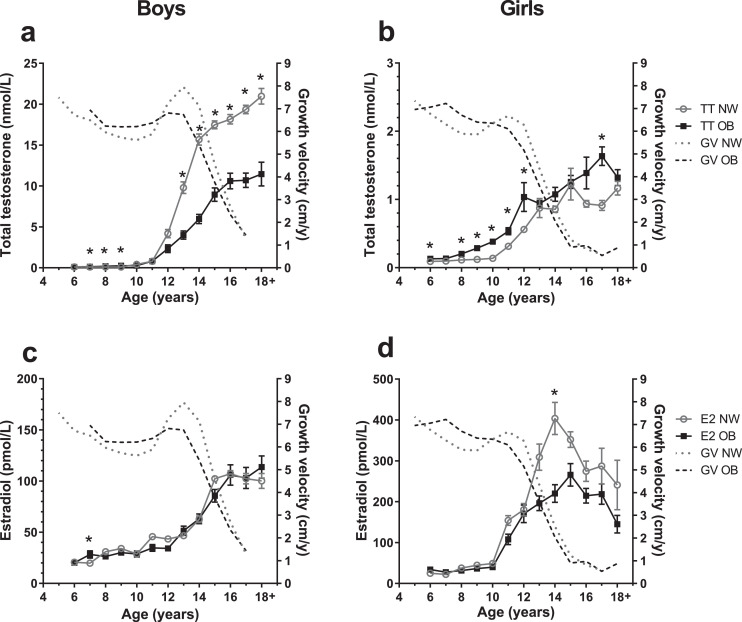
**Sex hormone levels of children with normal-weight and obesity**. Serum sex hormone levels of with obesity (OB, full black squares) and normal-weight (NW, open gray circles) groups of boys and girls are presented for age groups 5–18+ years. Data are shown as mean with standard error. The gray dotted-lined curves presents the growth velocities (GV) in cm per year (cm/y) of normal-weight individuals from whom sex steroid measurements were available. The black dashed-lined curves show growth velocities from individuals with obesity from sex steroids measurements were available. Growth velocities are presented as mean without standard error. Sample sizes of the sex steroid subcohort are provided in Additional file 1, Ie. a, b: Serum testosterone (TT) in nmol/L; c, d: Serum estradiol (E2) in pmol/L. Asterisks (*) mark significant (*p*<0·05) differences between NW and OB groups assessed by multiple t-tests combined with a Holm-Šídák multiple comparison test.

Regarding sex steroid levels (Fig. 4), during puberty, we found testosterone to be reduced by up to 62% in boys with obesity compared to normal-weight boys, along with decreased LH levels (Additional file 1, VI, Figure S8). Pubertal girls with obesity, in contrast, presented higher testosterone levels, but up to 37% reduced estradiol serum levels in late puberty (Fig. 4d). FSH levels were similar between normal-weight and with obesity groups, while DHEA-S was increased in prepubertal children with obesity (Additional file 1, VI, Figure S8). SHBG and FT4 were reduced, and TSH tended to be elevated in children with obesity constantly throughout childhood and adolescence (Additional file 1, VI, Figure S9).

During puberty, differences in growth velocity between normal-weight and with obesity groups corresponded to differences in testosterone levels in boys and in estradiol in girls and resembled the pattern of the IGF-1 levels. We did not observe any obvious association with growth velocity for FSH, SHBG, DHEA-S, TSH, or FT4 (Additional file 1, VI, Figure S8+9).

**Fig. 5:**
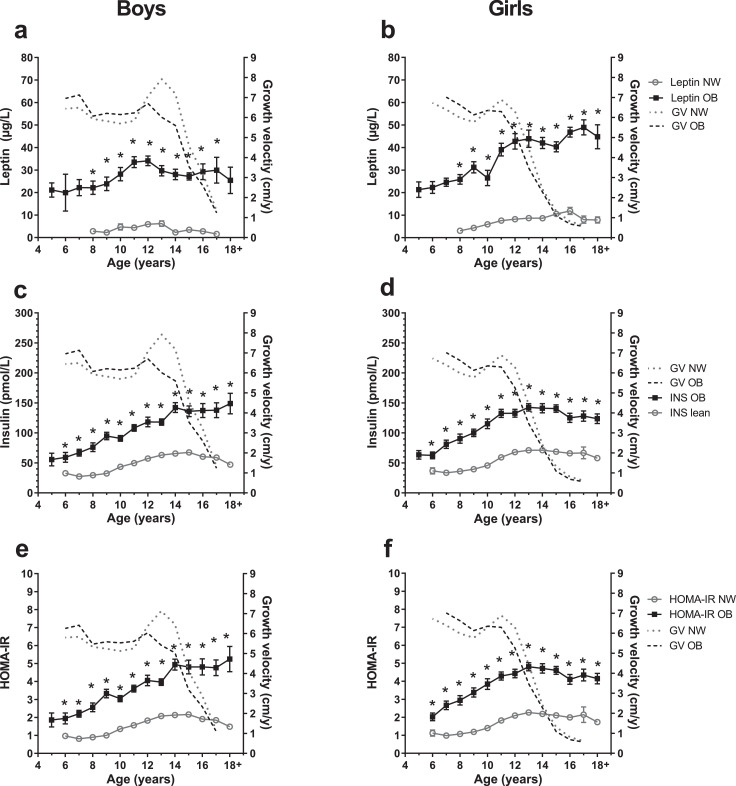
**Metabolic parameters of children with normal-weight and obesity.** Metabolic parameters of with obesity (OB, full black squares) and normal-weight (NW, open gray circles) boys and girls are depicted for age groups 5–18+ years. Data are shown as mean with standard error. The gray dotted-lined curves presents the growth velocities (GV) in cm per year (cm/y) of normal-weight individuals from whom metabolic measurements were available. The black dashed-lined curves show growth velocities from individuals with obesity from metabolic measurements were available. Growth velocities are presented as mean without standard error. Sample sizes of the metabolic factor subcohort are provided in Additional file 1, Ie. a, b: Serum leptin in µg/L; c, d: Serum fasting insulin in pmol/L. e, f: Homeostatic Model Assessment for Insulin Resistance (HOMA-IR). Asterisks (*) mark significant (*p*<0·05) differences between NW and OB children assessed by multiple t-tests combined with a Holm-Šídák multiple comparison test.

Serum leptin, insulin, and HOMA-IR were significantly elevated in with obesity compared to normal-weight groups during childhood. However, there was no particular similarity to the pattern of growth or growth velocity (Fig. 5).

### 3.6 Height, growth and endocrine factors in underweight children

The group of underweight children was shorter before puberty, followed by catch-up growth and finally reaching similar heights as the other weight groups (Additional file 1, VII). This delayed pubertal growth spurt was even more apparent when analyzing growth velocities and was accompanied by lower levels of IGF-1. Sex steroid levels also appeared slightly lower than in normal-weight children, whereas SHBG levels were higher showing the most distinct dependence on weight group. Hence, the observed effects of weight on growth dynamics and hormones were consistent across the weight categories, with underweight children presenting a relative delay in growth and pubertal development.

### 3.7 Reference values for children with obesity

**Fig. 6:**
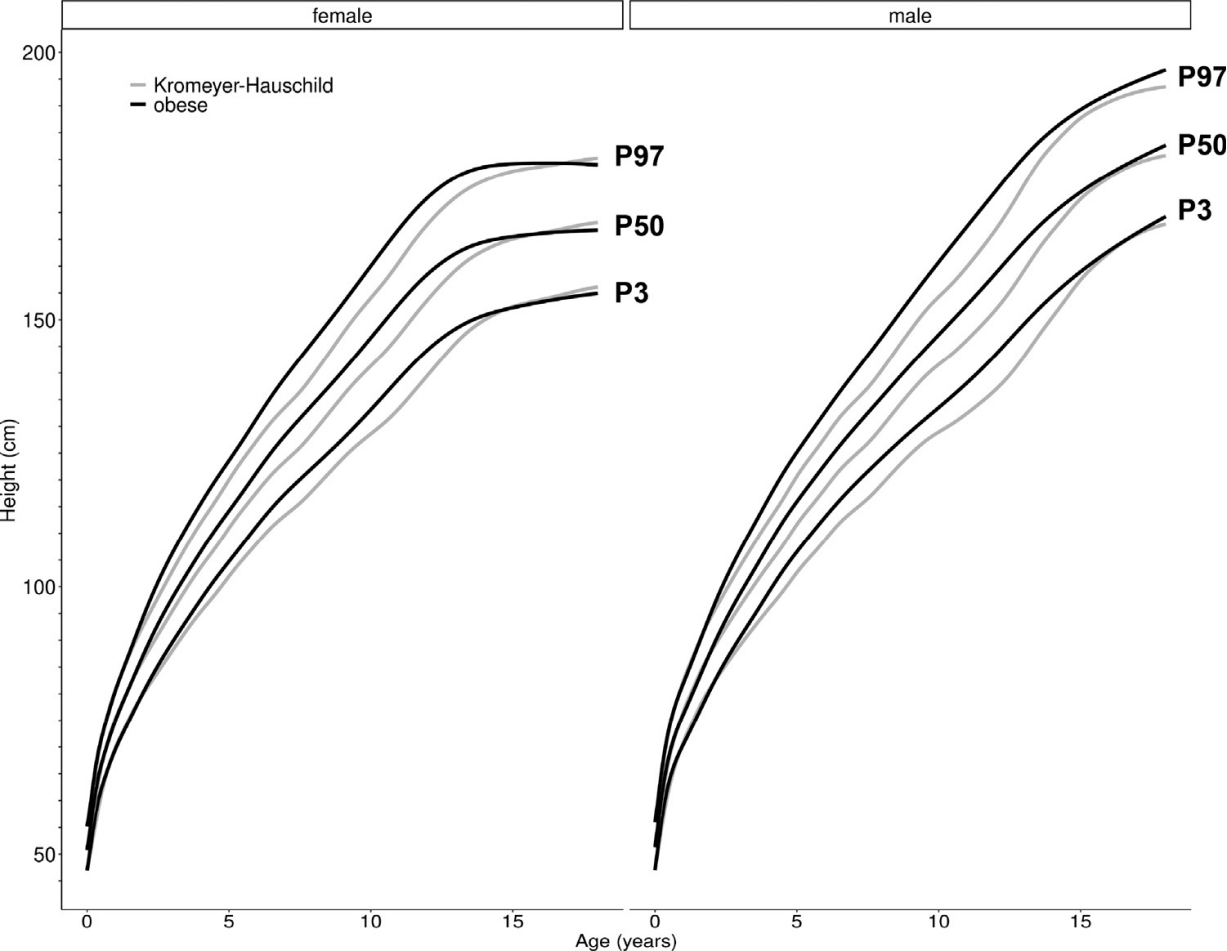
**Height percentiles for children with obesity compared to population-based reference values.** Height percentile curves in cm for children with obesity (BMI SDS ≥1.88) for boys and girls from ages 0 to 18 years derived from the CrescNet registry are presented (black lines) and compared to the height reference values according to Kromeyer-Hauschild [21] (gray lines). For each the 3rd (P3), the 50th (P50) and the 97th (P97) height percentiles are presented.

As growth curves of children with obesity, strongly differ from those of normal-weight children we need specific height reference values for children with obesity. Using data from a second, independent population-based study sample of *n* = 12,703, the CrescNet registry [2,24], we generated height curves for the 3rd, the 50th and the 97th percentile for boys and girls with obesity (Fig. 6). Corresponding height reference values have been estimated and are provided in Additional file 2. Height curves deviate from the percentile curves from the German references by Kromeyer-Hauschild et al. [21], which represent mostly normal-weight children and are comparable to the height curves of our normal-weight children of the CrescNet registry. As shown in our LIFE Child and Leipzig Childhood Obesity cohorts, children with obesity were taller until puberty, the pubertal growth spurt appeared to be blunted, hence reassuringly confirming the alterations in growth patterns in an independent populational cohort. The novel reference values are available open-access at the CrescNet website (Additional file 1, VIII, Figure S16) [32], the Ped(Z) Pediatric Calculator app (Additional file 1, VIII, Figure S17) [33] and via an R package [34].

## 4 Discussion

We here show that growth patterns of children with obesity deviate significantly and relevantly from those of normal-weight children with distinct dynamics across age. In early childhood, children with obesity are taller, independent of familial predisposition or increased birth length, whereas the pubertal growth spurt is blunted with concurrent decrease of growth velocity leading to a convergence of height curves in adolescence. The obesity-related alterations in growth velocity were confirmed by positive effects of BMI SDS on height SDS in the following year of age in early childhood and negative effects during puberty and are in line with recent results by Aris et al. [35] After completion of linear growth, children with obesity had a normal height around 0 SDS similar to normal-weight children. A recent study [7] reported compromised adult height in relation to mid-parental height in individuals with obesity. We found rather increased final height when corrected for mid-parental height. Those discrepancies might derive from the fact, that in the previous study the parents of the subjects with obesity tended to be taller than those from normal-weight children, while in our study parents of children with obesity were slightly shorter.

We confirmed the differences in height in children with obesity in a second, independent and population-based cohort, implying a high generalizability of the results on the population.

The excess height in states of obesity in early childhood develops upon slightly increased birth length, which may imply an early predisposition for accelerated growth right from (or before) birth. Thereafter, the observed obesity-related acceleration in growth velocity of up to 1 cm/year potentiates these differences accumulating to 7–8 cm taller height in children with obesity. This growth acceleration may be driven by growth factors, such as IGF-1 that is closely related to growth [12] and suspected to be dysregulated in obesity [12], [13], [14]. Looking into the dynamics of IGF-1 serum levels from early childhood to adulthood, we found IGF-1 to be increased in prepubertal children with obesity, although not to a great extent, while during puberty IGF-1 levels are decreased compared to normal-weight children paralleling the dynamics of growth velocities. Unfortunately, our sample size of IGF-1 in children younger than the age of 6 years was too low for the group with obesity to perform more detailed statistical analyses in order to determine the associations of increased IGF-1 on growth in early childhood.

Altered IGF-1 levels in obesity may be a consequence of an increased nutritional status and/or a general dysregulation involving various other hormones [9]. Indeed, we found parallel divergences in growth velocity and sex hormones between with obesity and normal-weight groups during puberty in line with previous studies showing that testosterone and gonadotropins were lower in pubertal boys with obesity [10,36]. Girls with obesity, in contrast, had an earlier onset and completion of puberty than normal-weight girls reflected by earlier thelarche and also by earlier onset of menarche [10]. Nevertheless, this is not likely to explain the alterations in early growth dynamics, as girls with obesity presented accelerated growth well before puberty onset. In line with Biro et al. [37], estradiol levels were reduced in our peripubertal girls with obesity and corresponding with deceleration of growth.

Girls with obesity had increased testosterone levels from 8 years onwards resulting in relative hyperandrogenism, particularly when the lower SHBG [36] levels in obesity are considered. The hyperandrogenism linked to obesity [10] somehow resembles polycystic ovary syndrome, but again is unlikely to be the reason for the generally increased testosterone levels in girls with obesity that have also been reported in prepubertal children and were reversible with weight loss [38].

It is known that sex steroids can modulate the GH/IGF-1 axis [16], for example by intensifying the amplitudes of GH pulses, by augmenting IGF-1 levels [39], or by increasing GH receptor expression and thereby potentially enhancing GH sensitivity [16]. The low testosterone levels in boys with obesity during puberty may hence contribute to the attenuated peak of serum IGF-1 and the lack of the growth spurt. However, this does not apply to girls with similarly repressed pubertal IGF-1 levels but elevated testosterone levels (even though they do not reach the extent of male testosterone) and hence does not suffice as an explanation for blunted pubertal IGF-1 response.

Finally, the elevated insulin and leptin levels in children with obesity [40] might promote growth. Insulin has receptor cross-reactivity for IGF-1 [9] and elevated insulin was associated with catch-up growth in infants born SGA [41]. Furthermore, hyperinsulinemia may be even primary to gigantism as observed in mice (not shown). Leptin may affect bone metabolism via direct molecular interaction in growth plates [9] and thereby stimulating proliferation and differentiation of chondrocytes [9].

We saw substantially elevated levels of metabolic and growth factors already in early childhood (even though still within normal reference ranges). The magnitude of the elevation in insulin and leptin levels further increased with age, hence not exactly paralleling the altered growth in childhood obesity.

Given the extensive impact of obesity at the populational level, our findings of accelerated linear growth in children with obesity during early childhood and the generation of reference values for height for children with obesity may have several implications. First, considering that growth is an important indicator of health on an individual level of health surveillance, a young child with obesity is expected to be relatively tall. Using the height reference values might avoid misinterpretation of tall stature with obesity and potentially unnecessary medical diagnostic work-up. On the other hand, relative short stature in a child with obesity may indicate underlying syndromic, (mono)genetic or another physical condition, which might be overlooked when applying reference values not tailored for the weight status. With the advance of modern genetic testing methods and potential treatment opportunities, early consideration and diagnosis of underlying disease cases may have direct and beneficial consequences for potential treatment [42], [43], [44]. Hence, the height reference values specific for children with obesity provide additional information for assessment of growth, but they are not intended to “normalize” the altered growth observed in children with obesity.

Second, our findings illustrate and add alterations in growth and endocrine factors to the array of obesity-related complications emerging already early in life, even though we cannot discern the direct mode and mechanism in this observational study. Finally, considering that early increased height gain was associated with higher adult cardiovascular risk [4,5], this puts children with obesity-related accelerated growth at increased risk for adult morbidity and potentially mortality [1].

There are several limitations of the study. First, this is a retrospective study over a long period combining cross-sectional and longitudinal data. Follow-up data allowing to calculate growth velocities were available in app. half the children. Likewise, endocrine parameters were only available in a subset of the cohort, which may limit the generalizability of the conclusions. Nevertheless, as cross-sectional data such as height, parental or endocrine data add important value to this study, we did not restrict the analyses to children with multiple visits. Furthermore, reassuringly these subsets showed identical growth patterns as the entire study population. Second, despite, standardized procedures for anthropometric measurements an inter-operator inaccuracy cannot be fully excluded, but might be compensated by high sample sizes and also applies to both, the normal-weight and with obesity groups. Height data of infants was not adjusted for gestational age. Third, we categorized children according to their weight status using the BMI. Although BMI is not a direct reflection of body fat, BMI is the most widely used index for the assessment of obesity with the advantage of being easily recordable and existing age, sex and often ethnic-specific reference values. In this study, we categorized children into normal-weight or with obesity according to the current weight status of the measurement, hence a change in weight category in follow-up analyses would not be regarded. However, for children, who have obesity in early childhood, the likelihood that obesity persists into adulthood is >80% [2]. Consistent with this assumption, in our study very few children changed from the normal-weight to the category with obesity (0.5%) and vice versa (2.5%).

Naturally, from this observational study we can only speculate about the mechanisms behind alterations in growth patterns with childhood obesity. It should be noted that the height reference values for children with obesity were generated from a German study sample; applying population-specific, national reference values may be recommended for other ethnic backgrounds.

In conclusion, growth patterns of children with obesity deviate from those of the normal-weight peer group throughout childhood and adolescence although the directions are temporarily distinct.

In early childhood obesity-related imbalances in the growth hormone axis and metabolic parameters may contribute to significantly increased growth and height in children with obesity. Also, the emergence of alterations in growth in early childhood before the manifestation of other profound sequelae indicates that predisposition, environmental and/or endocrine factors are modulating growth in children with obesity. During pubertal age, alterations in serum levels of the sex and growth hormone axis might contribute to the deceleration of growth in children with obesity. The provided novel height reference values for children with obesity may enhance the precision of individual clinical health surveillance.

## Funding

This work was supported by the German Research Foundation (DFG) for the Clinical Research Center “Obesity Mechanisms” SFB1052/CRC1052 (No. 209933838) project C05 to AK and LIFE – Leipzig Research Center for Civilization Diseases, University of Leipzig. LIFE is funded by means of the European Union, by means of the European Social Fund (ESF), by the European Regional Development Fund (ERDF), and by means of the Free State of Saxony within the framework of the excellence initiative. The project was further supported by the German Diabetes Association (DDG) and by the German Federal Ministry of Education and Research for the Integrated Research and Treatment Center Adiposity Diseases (Grant 01EO1501), and for the project SUCCEED (Grant 01GL1906). The CrescNet registry is supported by grants from Hexal Germany, Novo Nordisk Pharma, and Pfizer Pharma.

## Data sharing

De-identified data collected for this study, a data dictionary and statistical analysis plan are available from the corresponding author on reasonable request. Aggregated data are included in the supplementary files of this article. In addition, with publication, the novel obesity-specific height reference ranges are applicable for practical/clinical use at the CrescNet website [32], the Ped(Z) Pediatric Calculator app [33], and via an R package [34]

## Contributors

AK was the project principal investigator and led the design of the study, supervised statistical analyses, data interpretation. AK assumes responsibility for the completeness and integrity of the data and the fidelity of the report. WK established funding of LIFE child cohort, did project management, data interpretation and discussion of manuscript. EK, AK, MV, TV, JS, AKü, and KL performed data analyses, interpretation and drafted the manuscript. JK performed the laboratory measurements and participated in drafting of the manuscript. RG provided the CrescNet data. RP discussed the manuscript. ES was involved in recruiting participants of the study. DG implemented the reference values to the Ped(z) app. AK and EK have verified the underlying data. All authors contributed to the interpretation of study findings and had the opportunity to review and revise the final manuscript.

## Declaration of Competing Interest

Grants for supporting the CrescNet registry are reported by Ruth Gausche. Ruth Gausche reports grants from NovoNordik, grants from Hexal AG, grants from Pfizer, during the conduct of the study. Dr. Körner reports grants from LIFE Child, grants from Childhood Obesity cohort, during the conduct of the study; personal fees from Ipsen Pharma, personal fees from Novo Nordisk, for lecturing outside the submitted work. All the other authors report no conflicts.

## Appendix Supplementary materials

Image, application 1

Image, application 2

Image, application 3
