# Supplementary material for: Dynamic alterations in linear growth and endocrine parameters in children with obesity and height reference values
Source: eClinicalMedicine. 2021 Jun 23;37:100977. doi: 10.1016/j.eclinm.2021.100977 (PMC8343253; doi:10.1016/j.eclinm.2021.100977)
Supplement: Supplementary file 1 [file mmc1.docx]

**Caption for Supplementary Material**

1. Additional File 1: Additional information and analyses and available online tools for applying height reference values for children with obesity
2. Additional File 2: Reference values for height for boys and girls with obesity from ages 0-18 years for the 2.5th, 5th, 50th, 95th and 97.5th percentile.
