# Supplementary material for: Dynamic alterations in linear growth and endocrine parameters in children with obesity and height reference values
Source: eClinicalMedicine. 2021 Jun 23;37:100977. doi: 10.1016/j.eclinm.2021.100977 (PMC8343253; doi:10.1016/j.eclinm.2021.100977)
Supplement: Supplementary file 2 [file mmc2.docx]

**Additional file 1**

**Table of contents**

I. Study population and selection……………………………………………………………………………….….2

Ia. Description of the cohorts…………………………………………………………………….…….…2

Ib. Inclusion criteria for height SDS and BMI SDS (Figure S1)…………………………………………3

Ic. Children included in the analyses (Figure S2)………………………………………………..…….…4

Id. Characteristic of the cohorts (Table S1)…………………………………………………………..…..5

Ie. Numbers of observations per weight category and age group in the subcohorts (Table S2)………....7

If. Multiple observations (Table S3)………………………………………………………………...……9

II. The CrescNet registry

IIa. Description of the registry……………..……………………………………………………………10

IIb. Inclusion criteria for the generation of height reference values for children with obesity

(Figure S3)………………………………………………………………………………………………11

III. Pubertal stages (Table S4, Table S5)…………………………………………………………………………12

IV. Parental data and birth parameters……………………………………………………...……….……....……13

IVa. Parental height (Figure S4)………………………………………………...………….……..….…13

IVb. Birth parameters (Table S6, Figure S5)………………………………..………….…….……..…..14

IVc. Obesity-related increase in height in early childhood is independent from parental height or birth parameters (Figure S6, Table S7)…………………………………………………...…….….…………16

V. Non-parametric modelling of growth patterns and IGF-1 (Figure S7)………… ………………..……...……18

VI. Serum levels of additional hormones in children with normal-weight and obesity in relation to growth velocities (Figure S8, Figure S9) .....…………………………………………………………………….….……19

VII. Height and endocrine factors in children with underweight, normal-weight, overweight or obesity

(Figure S10-15)……..…….………………………………………..……………………………………………..21

VIII. Online tools: Height reference values for children with obesity (Figure S16-17)………….……………...27

Additional file 1 References…………………………………………………………………………...………….29

**I. Study population and selection**

**Ia. Description of the cohorts**

Our study encompassed 8,629 children with 37,493 observations across ages 0 to 20 years from the population-based LIFE Child[^1^](#_ENREF_1)^,^[^2^](#_ENREF_2) (44·3% of the children) and the obesity-enriched Leipzig Obesity Childhood Cohort[^3^](#_ENREF_3)^,^[^4^](#_ENREF_4) (55·7% of the children) recruited at the University Hospital for Children & Adolescents Leipzig in Germany from 1999 to 2018.

LIFE Child[^1^](#_ENREF_1)^,^[^2^](#_ENREF_2) is an ongoing regional population-based study with comprehensive phenotyping conducted in the city of Leipzig, Germany aiming to monitor healthy child development from birth to adulthood and to understand the development of civilization diseases. Participants undergo a comprehensive evaluation comprising of medical, psychological, and sociodemographic assessments as well as the collection of biological samples. With recruitment age ranging between the 24th week of gestation to 16 years of age and annual follow-ups, the study combines a cross-sectional with a longitudinal design and covers a broad age range. The study was approved by the ethics committee of the University of Leipzig (NCT02550236).

For the Leipzig Obesity Childhood Cohort[^3-5^](#_ENREF_3) patients with obesity were consecutively recruited from our outpatient obesity clinic. The study was approved by the ethics committee of the University of Leipzig (NCT04491344). Patients underwent metabolic testing along with medical and clinical assessments at the same study center. Hence, applied methodology for both cohorts was identical.

Written consent was obtained from parents and assent from children older than 12 years.

**Ib. Inclusion criteria for height SDS and BMI SDS**

In Figure S1 the rationales for the inclusion criteria for height standard deviation score (SDS) and body mass index (BMI) SDS are outlined. Children with height SDS between -2·5 and 4·0 and BMI SDS higher than -3·5 were included in the study based on the good fit in normal distribution plots.

**b**

**a**

**d**

**c**

**Figure S1. Children with height standard deviation score (SDS) between -2·5 and 4·0 and a body mass index (BMI) SDS higher than -3·5 were included in the study based on linearity in normal distribution plots**

Children with diseases, manifested clinically confirmed precocious puberty and medication were already excluded in those graphs. a, b: Normal distribution plots of height SDS for boys and girls; c, d: Normal distribution plots of BMI SDS for boys and girls.

**Ic. Children included in the analyses**

In Figure S2 the inclusion criteria for the study are presented step-wise in a flow chart:

Children aged 0 to 20 years with a height standard deviation score (SDS) from -2·5 to 4·0 and a body mass index SDS higher than -3·5 were initially included (Additional file 1, Ib). Children suffering from diseases such as type 1 diabetes (T1DM), syndromes and other conditions with severe disability (permanent immobility, microcephaly etc.) and children with manifested precocious puberty, intracranial hypertension or valproate medication were excluded, as well as individuals taking medications affecting growth (e.g. growth hormone, systemic glucocorticoids, immunosuppressives).

Exclusively for analyzing insulin and Homeostatic Model Assessment-Insulin Resistance (HOMA-IR) or thyroid hormones, data of children taking metformin or thyroxin, respectively, were excluded. Observations of children born prematurely (gestational week<38) were included for age at measurement ≥2·0 years as by then they are supposed to have caught up in height[^6^](#_ENREF_6) and height SDS did not differ significantly anymore between pre-term and full-term normal-weight children (data not shown).

In total 37,493 observations of 4,319 boys (**♂**) and 4,310 girls (**♀**) (total 8,629 children) were used for analyses. For analyzing height parameters and circulating factors only the first observation per individual per age group was included accounting altogether for 27,900 observations from the 8,629 children. Growth velocities of 3,269 children with 10,541 observations (first observation per individual per age group) were included. Growth velocities were only included when the mean age of a child between the two height observations was older than 1·5 years. A more detailed description of the numbers of observations for the different parameters is shown in Additional file 1, Id+e.


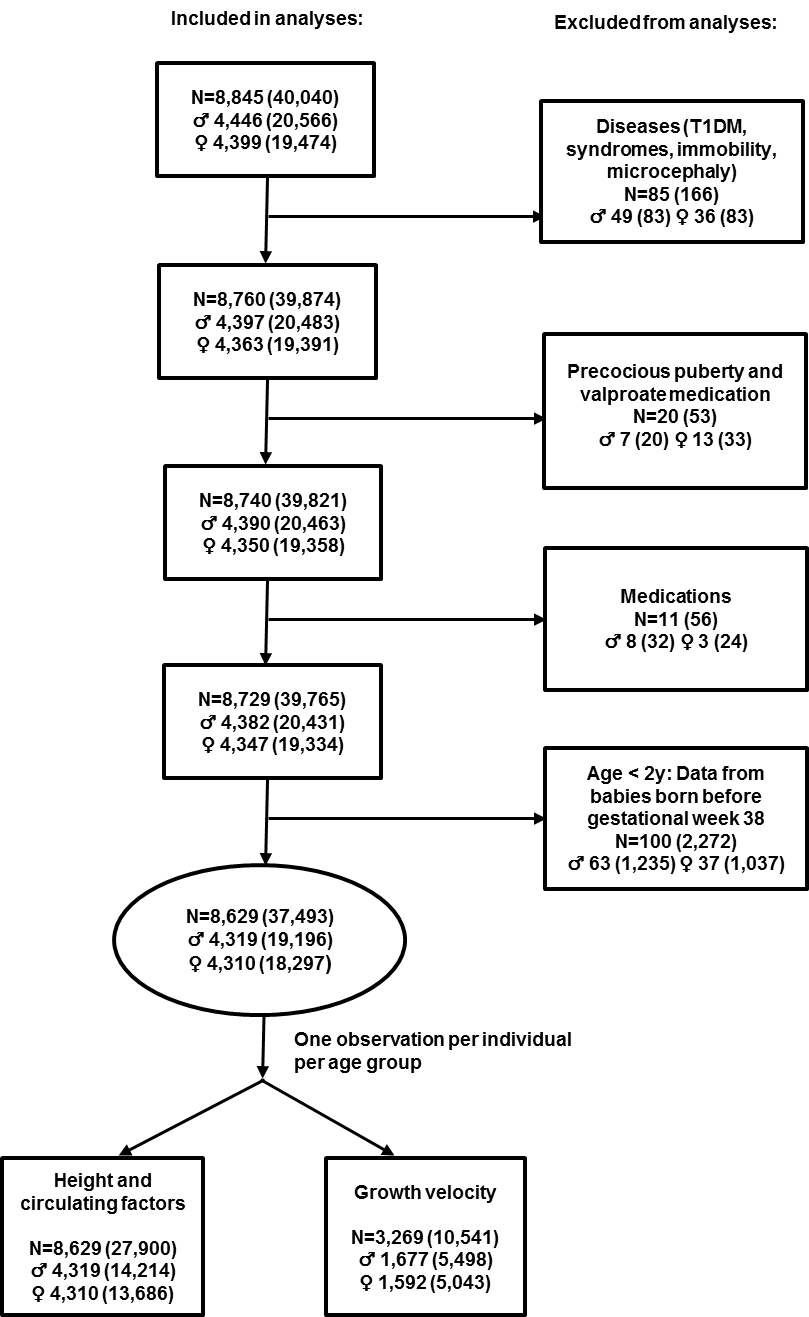


**Figure S2. Children included in the analyses**

Children or observations were excluded due to diseases, conditions of precocious puberty, medication or premature birth (until the age of 2 years). The number of subjects (N) and observations (shown in brackets) are indicated for all children/observations and for boys (**♂**) and girls (**♀**) separately.

**Id. Characteristics of the cohort**

**Table S1. Overview of the anthropometric and clinical data of the combined cohorts****.** Data sets were categorized according to sex and BMI SDS class. Data are presented as mean ± standard deviation (SD) and range (minimum and maximum values) and number of observations (N) are provided considering only one observation per individual per age class. Data for growth velocity are shown for children ≥1·5 years (mean age between the two height observations).

| **Weight category** |  | **UW** |  |  | **NW** |  |  | **OW** |  |  | **OB** |  |
| --- | --- | --- | --- | --- | --- | --- | --- | --- | --- | --- | --- | --- |
| **Boys** | **N** | **Mean (SD)** | **Range** | **N** | **Mean (SD)** | **Range** | **N** | **Mean (SD)** | **Range** | **N** | **Mean (SD)** | **Range** |
| **Age, years** | 953 | 6·9 ( 5·1) | 0 - 19·8 | 10,324 | 5·9 ( 5) | 0 - 20·3 | 1,129 | 6·4 ( 5·6) | 0 - 19 | 1,808 | 9·3 ( 5·3) | 0 - 20·3 |
| **Height, cm** | 953 | 117·5 ( 34·4) | 46 - 194·2 | 10,324 | 111·7 ( 37·2) | 46 - 200·5 | 1,129 | 113·8 ( 44·6) | 46 - 196·3 | 1,808 | 137·2 ( 41) | 47 - 201 |
| **Height SDS** | 953 | -0·2 ( 1) | -2·5 - 3·5 | 10,324 | 0·1 ( 0·9) | -2·5 - 3·9 | 1,129 | 0·3 ( 1) | -2·3 - 3·7 | 1,808 | 0·6 ( 1) | -2·5 - 3·9 |
| **BMI SDS** | 953 | -1·7 ( 0·4) | -3·4 - -1·3 | 10,324 | 0 ( 0·6) | -1·3 - 1·3 | 1,129 | 1·6 ( 0·2) | 1·3 - 1·9 | 1,808 | 2·5 ( 0·5) | 1·9 - 4·9 |
| **Height_parental adj_ SDS** | 559 | -0·2 ( 0·9) | -2·4 - 3·4 | 5,960 | 0·1 ( 0·9) | -2·5 - 4 | 656 | 0·4 ( 1) | -2·3 - 3·6 | 1,132 | 0·8 ( 1) | -2·3 - 3·8 |
| **Growth velocity, cm/y** | 402 | 6·7 (2·2) | 0·2 - 15·1 | 4,286 | 7·1 (2·3) | 0 - 21·9 | 341 | 7·1 (2·7) | 0 - 16·3 | 469 | 6·2 (2·6) | 0 - 15 |
| **Growth velocity SDS** | 402 | -0·2 (1·6) | -6·6 - 7 | 4,286 | 0·2 (1·5) | -6·9 - 9·8 | 341 | 0·3 (1·6) | -3·5 - 7·6 | 469 | 0·2 (1·5) | -3·5 - 9·1 |
| **TSH, mIU/L** | 386 | 2·7 (1·3) | 0·4 - 9·6 | 3,582 | 2·7 (1·3) | 0·3 - 14·9 | 365 | 2·7 (1·2) | 0·6 - 8·7 | 1,009 | 3 (1·3) | 0·4 - 8·9 |
| **FT4, pmol/L** | 374 | 16·1 (1·8) | 9·7 - 21·9 | 3,504 | 16·2 (2) | 8·8 - 45·4 | 358 | 15·3 (2·1) | 5·8 - 24·4 | 1,008 | 15·1 (2·2) | 2·8 - 24·8 |
| **LH, IU/L** | 318 | 1·5 ( 1·6) | 0·1 - 8·6 | 3,007 | 1·6 ( 1·8) | 0 - 10·9 | 346 | 1·7 ( 1·7) | 0 - 7·6 | 841 | 1·7 ( 2) | 0 - 25·1 |
| **FSH, IU/L** | 320 | 2·4 ( 1·9) | 0·1 - 10·6 | 3,007 | 2·3 ( 2) | 0·1 - 20 | 347 | 2·5 ( 1·6) | 0·2 - 9·2 | 847 | 2·4 ( 2) | 0 - 18·7 |
| **DHEA-S, µmol/L** | 138 | 3·1 (2·5) | 0·1 - 15·3 | 1,345 | 3·4 (3) | 0 - 20·3 | 136 | 4·8 (3) | 0·3 - 15·9 | 194 | 4·4 (2·7) | 0·2 - 16·3 |
| **Estradiol, pmol/L** | 191 | 50·8 (35·7) | 18·4 - 164·2 | 1,951 | 55·1 (40·6) | 18·4 - 259·9 | 232 | 57·6 (39·7) | 0 - 277·4 | 437 | 51·5 (39·6) | 0 - 200·3 |
| **Testosterone, nmol/L** | 136 | 6·2 (7·6) | 0·1 - 25 | 1,319 | 6·3 (8·6) | 0·1 - 35·5 | 180 | 6·1 (6·9) | 0·1 - 27 | 501 | 3·8 (5) | 0·1 - 21·8 |
| **SHBG, nmol/L** | 400 | 106·2 ( 50·9) | 1·2 - 284·8 | 3,878 | 98 ( 54·5) | 2·1 - 978·2 | 339 | 60·1 ( 44·1) | 5·8 - 222·1 | 381 | 41·1 ( 30·9) | 1·4 - 194 |
| **IGF-1, ng/mL** | 306 | 170·9 ( 117·4) | 21·3 - 722 | 2,695 | 195·6 ( 131·9) | 20 - 902 | 236 | 243·7 ( 134) | 34·2 - 717 | 392 | 236·1 ( 113·3) | 38·5 - 624 |
| **IGFBP-3, µg/mL** | 283 | 3·8 ( 1·2) | 1·3 - 8·2 | 2,522 | 4 ( 1·1) | 1·2 - 7·8 | 213 | 4·4 ( 1·1) | 1·3 - 7·9 | 372 | 4·5 ( 1) | 1·8 - 8 |
| **Leptin, ng/mL** | 38 | 1·8 (1·2) | 0·2 - 4·3 | 265 | 3·5 (3·8) | 0·2 - 23·7 | 63 | 13·5 (11·7) | 0·2 - 60·9 | 343 | 28·4 (15) | 0·2 - 88·5 |
| **Insulin, pmol/L** | 138 | 40·7 (21) | 5·1 - 113·3 | 1,288 | 49 (27·1) | 3·6 - 238·2 | 232 | 79·2 (47) | 4·7 - 518·1 | 956 | 111·9 (72·3) | 5 - 571·7 |
| **HOMA-IR** | 133 | 1·3 (0·7) | 0·2 - 3·9 | 1,261 | 1·6 (0·9) | 0·1 - 8·4 | 227 | 2·7 (1·7) | 0·1 - 15·7 | 950 | 3·8 (2·7) | 0·1 - 28·8 |

UW - underweight; NW- normal-weight; OW - overweight; OB - obese; BMI - Body mass index; SDS - standard deviation score; Height_parental adj_ SDS - Height SDS relative to mid-parental height SDS; TSH - thyroid-stimulating hormone; FT4 - free thyroxin; LH - luteinizing hormone; FSH - follicle-stimulating hormone; DHEA-S - dehydroepiandrosterone sulfate; SHBG - sex hormone-binding globulin; IGF-1 - insulin-like growth factor-1; IGFBP-3 – IGF-binding protein 3; HOMA-IR - homeostatic model assessment for insulin resistance.

**Table S1. Overview of the anthropometric and clinical data of the combined cohorts (continued)** Data sets were categorized according to sex and BMI SDS class. Data are presented as mean ± standard deviation (SD) and range (minimum and maximum values) and number of observations (N) are provided considering only one observation per individual per age class. Data for growth velocity are shown for children ≥older than 1·5 years (mean age between the two height observations).

| **Weight category** |  | **UW** |  |  | **NW** |  |  | **OW** |  |  | **OB** |  |
| --- | --- | --- | --- | --- | --- | --- | --- | --- | --- | --- | --- | --- |
| **Girls** | **N** | **Mean (SD)** | **Range** | **N** | **Mean (SD)** | **Range** | **N** | **Mean (SD)** | **Range** | **N** | **Mean (SD)** | **Range** |
| **Age, years** | 814 | 6·7 ( 5·1) | 0 - 19·8 | 9,665 | 6·1 ( 5·1) | 0 - 19·8 | 1,151 | 6·5 ( 5·6) | 0 - 18·9 | 2,056 | 9·7 ( 5·6) | 0 - 20 |
| **Menarche, y** | 88 | 13 (1·2) | 10 - 16 | 1,146 | 12·5 (1·1) | 9 - 16 | 175 | 12·2 (1·1) | 9 - 16 | 476 | 11·8 (1·3) | 9 - 16 |
| **Height, cm** | 814 | 115·3 ( 34·7) | 46 - 182·8 | 9,665 | 110·7 ( 36·2) | 46 - 185·7 | 1,151 | 113·1 ( 43·1) | 46 - 189·5 | 2,056 | 135·7 ( 39·2) | 46 - 187·8 |
| **Height SDS** | 814 | -0·2 ( 1) | -2·5 - 3·4 | 9,665 | 0 ( 0·9) | -2·5 - 3·7 | 1,151 | 0·3 ( 1·1) | -2·4 - 3·9 | 2,056 | 0·5 ( 1·1) | -2·5 - 3·9 |
| **BMI SDS** | 814 | -1·7 ( 0·4) | -3·5 - -1·3 | 9,665 | 0 ( 0·7) | -1·3 - 1·3 | 1,151 | 1·6 ( 0·2) | 1·3 - 1·9 | 2,056 | 2·6 ( 0·6) | 1·9 - 5·9 |
| **Height_parental adj_ SDS** | 450 | 0 ( 0·9) | -2·5 - 2·9 | 5,686 | 0·1 ( 0·9) | -2·5 - 3·6 | 690 | 0·4 ( 1·1) | -2·4 - 3·4 | 1,209 | 0·8 ( 1·1) | -2·4 - 3·9 |
| **Growth velocity, cm/y** | 318 | 6·2 (2·4) | 0 - 18·4 | 3,856 | 6·8 (2·7) | 0 - 20·2 | 332 | 6·6 (3·3) | 0 - 17·1 | 537 | 4·8 (3·3) | 0 - 15·6 |
| **Growth velocity SDS** | 318 | -0·2 (1·8) | -7 - 5 | 3,856 | 0·2 (1·7) | -7·5 - 9·1 | 332 | 0·3 (1·8) | -5·8 - 9 | 537 | 0 (1·9) | -5·6 - 8·8 |
| **TSH, mIU/L** | 274 | 2·5 (1·2) | 0·6 - 8 | 3,138 | 2·6 (1·2) | 0·1 - 10·8 | 361 | 2·8 (1·3) | 0·5 - 8·3 | 1,170 | 2·9 (1·4) | 0 - 14·4 |
| **FT4, pmol/L** | 268 | 16·1 (2·1) | 11·3 - 23·5 | 3,083 | 16·1 (2·1) | 7·2 - 40·4 | 352 | 15·6 (2·5) | 6·6 - 37·9 | 1,168 | 15·2 (2·3) | 5·4 - 32·8 |
| **LH, IU/L** | 242 | 2·8 ( 5·2) | 0 - 46·6 | 2,766 | 3·2 ( 6·1) | 0 - 98·8 | 372 | 3·5 ( 5·2) | 0 - 43·9 | 1,009 | 3·9 ( 5·3) | 0 - 58·4 |
| **FSH, IU/L** | 243 | 3·9 ( 2·4) | 0·1 - 11·4 | 2,765 | 3·7 ( 2·5) | 0·1 - 21·7 | 371 | 3·6 ( 2·3) | 0·1 - 11·8 | 1,016 | 3·7 ( 2·5) | 0 - 21·3 |
| **DHEA-S, µmol/L** | 119 | 2·8 (2·4) | 0·1 - 17·4 | 1,215 | 3·1 (2·5) | 0 - 18·3 | 128 | 3·7 (2·1) | 0·4 - 10·5 | 215 | 4 (2·4) | 0·2 - 11·5 |
| **Estradiol, pmol/L** | 165 | 134·8 (186·1) | 18·4 - 1359 | 1,869 | 186·7 (274·3) | 18·4 - 2518 | 306 | 163·8 (220·3) | 18·4 - 1452 | 874 | 145·9 (176·3) | 0 - 1649·3 |
| **Testosterone, nmol/L** | 119 | 0·5 (0·6) | 0·1 - 3·8 | 1,190 | 0·5 (1) | 0·1 - 17·5 | 178 | 0·6 (0·5) | 0·1 - 2·5 | 596 | 0·9 (1) | 0·1 - 14·7 |
| **SHBG, nmol/L** | 284 | 114·4 ( 56·9) | 2·7 - 448·7 | 3,515 | 100·7 ( 52·8) | 1·4 - 488·2 | 344 | 69·3 ( 54·4) | 4·7 - 336·6 | 421 | 45·6 ( 43·1) | 1·7 - 290·8 |
| **IGF-1, ng/mL** | 222 | 202 ( 116·6) | 26·6 - 585 | 2,391 | 228·3 ( 132·8) | 21·4 - 888 | 237 | 283·8 ( 141·2) | 25·5 - 786 | 406 | 272·1 ( 114·6) | 53 - 695 |
| **IGFBP-3, µg/mL** | 209 | 4·2 ( 1·1) | 1·4 - 7 | 2,195 | 4·2 ( 1·1) | 1·3 - 8·4 | 211 | 4·5 ( 1) | 1·6 - 7·5 | 398 | 4·7 ( 0·9) | 2 - 9·4 |
| **Leptin, ng/mL** | 28 | 3·9 (2·7) | 0·2 - 9·6 | 295 | 7·6 (5·4) | 0·2 - 35·2 | 70 | 18·7 (9·7) | 3·1 - 53 | 390 | 38·3 (18·4) | 0·2 - 109 |
| **Insulin, pmol/L** | 128 | 48·5 (25) | 11·1 - 179·8 | 1,242 | 56·7 (33·9) | 4·2 - 722·9 | 240 | 84·7 (39·4) | 10·4 - 231·6 | 1,121 | 120·4 (70·4) | 3·5 - 578·3 |
| **HOMA-IR** | 126 | 1·5 (0·8) | 0·3 - 4·9 | 1,218 | 1·7 (1·2) | 0·1 - 30·7 | 238 | 2·7 (1·4) | 0·4 - 7·7 | 1,118 | 4 (2·5) | 0·1 - 19·7 |

UW - underweight; NW- normal-weight; OW - overweight; OB - obese; BMI - Body mass index; SDS - standard deviation score; Height_parental adj_ SDS - Height SDS relative to mid-parental height SDS; TSH - thyroid-stimulating hormone; FT4 - free thyroxin; LH - luteinizing hormone; FSH - follicle-stimulating hormone; DHEA-S - dehydroepiandrosterone sulfate; SHBG - sex hormone-binding globulin; IGF-1 - insulin-like growth factor-1; IGFBP-3 - IGF-binding protein 3; HOMA-IR - homeostatic model assessment for insulin resistance.

**Ie. Numbers of observations per weight category and age group in the subcohorts**

**Table S2. Numbers of observations per weight category and age group for the entire cohort (height) and subcohorts for growth velocity, IGF-1 or IGFBP-3, sex steroids, metabolic factors and thyroid hormones** Height parameters were available for the entire cohort. Subcohorts were built for observations with growth velocity (children ≥ mean age 1·5 years), IGF-1 or IGFBP-3 measurements, sex steroid (FSH, LH, DHEA-S, SHBG, estradiol or testosterone), metabolic factor (leptin, insulin or HOMA-IR) and thyroid hormone (TSH or FT4) measurements. As also in Table S1 (Additional file 1, Id) only one observation per individual per age class was included.

| **Boys** | **Height (entire cohort)** | | | | **Growth velocity** | | | | **IGF-1 or IGFBP-3** | | | | **Sex steroids** | | | | **Metabolic factors** | | | | **Thyroid hormones** | | | |
| --- | --- | --- | --- | --- | --- | --- | --- | --- | --- | --- | --- | --- | --- | --- | --- | --- | --- | --- | --- | --- | --- | --- | --- | --- |
| **age** | **UW** | **NW** | **OW** | **OB** | **UW** | **NW** | **OW** | **OB** | **UW** | **NW** | **OW** | **OB** | **UW** | **NW** | **OW** | **OB** | **UW** | **NW** | **OW** | **OB** | **UW** | **NW** | **OW** | **OB** |
| **0** | 40 | 1,160 | 223 | 207 | - | - | - | - | 8 | 69 | 9 | 3 | 0 | 0 | 0 | 0 | 0 | 1 | 0 | 0 | 15 | 171 | 13 | 6 |
| **1** | 139 | 1,216 | 104 | 59 | - | - | - | - | 18 | 71 | 3 | 0 | 24 | 130 | 5 | 2 | 0 | 1 | 0 | 1 | 27 | 156 | 8 | 3 |
| **2** | 92 | 1,179 | 113 | 58 | 25 | 320 | 20 | 9 | 9 | 88 | 6 | 0 | 10 | 145 | 15 | 2 | 1 | 5 | 0 | 0 | 11 | 164 | 16 | 3 |
| **3** | 34 | 550 | 47 | 21 | 48 | 642 | 51 | 35 | 9 | 130 | 10 | 2 | 7 | 167 | 13 | 5 | 2 | 6 | 1 | 4 | 11 | 188 | 14 | 7 |
| **4** | 97 | 1,104 | 73 | 62 | 28 | 473 | 29 | 21 | 5 | 112 | 3 | 6 | 8 | 160 | 8 | 7 | 0 | 4 | 2 | 7 | 10 | 171 | 9 | 9 |
| **5** | 88 | 968 | 65 | 95 | 71 | 754 | 55 | 33 | 12 | 129 | 6 | 2 | 15 | 175 | 7 | 11 | 1 | 5 | 1 | 16 | 21 | 198 | 8 | 17 |
| **6** | 24 | 295 | 9 | 36 | 26 | 298 | 15 | 16 | 11 | 154 | 5 | 11 | 12 | 188 | 6 | 26 | 5 | 66 | 1 | 26 | 14 | 206 | 7 | 27 |
| **7** | 28 | 279 | 14 | 56 | 14 | 180 | 4 | 16 | 14 | 146 | 6 | 12 | 17 | 181 | 8 | 36 | 4 | 78 | 2 | 42 | 18 | 206 | 9 | 45 |
| **8** | 45 | 411 | 28 | 82 | 17 | 199 | 11 | 18 | 14 | 191 | 8 | 23 | 25 | 307 | 15 | 58 | 8 | 121 | 9 | 54 | 23 | 244 | 15 | 61 |
| **9** | 45 | 510 | 43 | 96 | 21 | 216 | 11 | 28 | 22 | 222 | 10 | 39 | 33 | 393 | 29 | 77 | 11 | 133 | 16 | 66 | 26 | 262 | 17 | 64 |
| **10** | 37 | 345 | 44 | 124 | 20 | 200 | 17 | 37 | 24 | 200 | 21 | 35 | 27 | 269 | 37 | 91 | 8 | 144 | 23 | 98 | 26 | 254 | 29 | 95 |
| **11** | 55 | 485 | 75 | 141 | 23 | 196 | 17 | 26 | 32 | 206 | 21 | 36 | 42 | 367 | 53 | 96 | 13 | 134 | 41 | 107 | 34 | 240 | 39 | 100 |
| **12** | 42 | 374 | 68 | 135 | 26 | 186 | 25 | 48 | 22 | 212 | 35 | 46 | 31 | 296 | 55 | 96 | 17 | 147 | 39 | 105 | 28 | 242 | 47 | 99 |
| **13** | 50 | 276 | 63 | 182 | 27 | 171 | 27 | 59 | 28 | 184 | 28 | 56 | 35 | 225 | 54 | 135 | 16 | 132 | 43 | 141 | 38 | 215 | 48 | 145 |
| **14** | 42 | 267 | 54 | 148 | 21 | 163 | 24 | 40 | 25 | 164 | 19 | 56 | 34 | 206 | 42 | 104 | 21 | 123 | 33 | 109 | 31 | 213 | 38 | 116 |
| **15** | 47 | 443 | 52 | 129 | 15 | 143 | 19 | 45 | 27 | 175 | 20 | 30 | 44 | 366 | 36 | 78 | 28 | 150 | 20 | 99 | 25 | 173 | 17 | 90 |
| **16** | 28 | 276 | 31 | 88 | 13 | 94 | 13 | 23 | 17 | 149 | 19 | 23 | 24 | 219 | 27 | 62 | 15 | 111 | 14 | 60 | 15 | 158 | 18 | 59 |
| **17** | 14 | 128 | 13 | 56 | 6 | 49 | 3 | 14 | 7 | 78 | 8 | 10 | 10 | 110 | 10 | 43 | 5 | 62 | 5 | 41 | 10 | 87 | 7 | 43 |
| **18+** | 6 | 58 | 10 | 33 | 1 | 2 | 0 | 1 | 3 | 27 | 1 | 3 | 5 | 44 | 7 | 27 | 3 | 38 | 7 | 29 | 2 | 35 | 6 | 25 |
| **Total** | 953 | 10,324 | 1,129 | 1,808 | 402 | 4,286 | 341 | 469 | 307 | 2,707 | 238 | 393 | 403 | 3,948 | 427 | 956 | 158 | 1,460 | 257 | 1,005 | 385 | 3,583 | 365 | 1,014 |

UW - underweight; NW- normal-weight; OW - overweight; OB - obese; IGF-1 - insulin-like growth factor-1; IGFBP-3 - IGF-binding protein 3; FSH - follicle-stimulating hormone; LH - luteinizing hormone; DHEA-S - dehydroepiandrosterone sulfate; SHBG - sex hormone-binding globulin; HOMA-IR - homeostatic model assessment for insulin resistance; TSH - thyroid-stimulating hormone; FT4 - free thyroxin.

**Table S2. Numbers of observations per weight category and age group for the entire cohort (height) and subcohorts for growth velocity, IGF-1 or IGFBP-3, sex steroids, metabolic factors and thyroid hormones (continued)** Height parameters were available for the entire cohort. Subcohorts were built for observations with growth velocity (children ≥ mean age 1·5 years), IGF-1 or IGFBP-3 measurements, sex steroid (FSH, LH, DHEA-S, SHBG, estradiol or testosterone), metabolic factor (Leptin, Insulin or HOMA-IR) and thyroid hormone (TSH or FT4) measurements. As also in Table S1 (Additional file 1, Id) only one observation per individual per age class was included.

| **Girls** | **Height (entire cohort)** | | | | **Growth velocity** | | | | **IGF-1 or IGFBP-3** | | | | **Sex steroids** | | | | **Metabolic factors** | | | | **Thyroid hormones** | | | |
| --- | --- | --- | --- | --- | --- | --- | --- | --- | --- | --- | --- | --- | --- | --- | --- | --- | --- | --- | --- | --- | --- | --- | --- | --- |
| **age** | **UW** | **NW** | **OW** | **OB** | **UW** | **NW** | **OW** | **OB** | **UW** | **NW** | **OW** | **OB** | **UW** | **NW** | **OW** | **OB** | **UW** | **NW** | **OW** | **OB** | **UW** | **NW** | **OW** | **OB** |
| **0** | 44 | 1,038 | 216 | 224 | - | - | - | - | 6 | 58 | 2 | 4 | 0 | 0 | 0 | 0 | 0 | 0 | 0 | 0 | 10 | 136 | 15 | 3 |
| **1** | 118 | 1,112 | 107 | 77 | - | - | - | - | 10 | 53 | 4 | 0 | 13 | 106 | 5 | 0 | 0 | 0 | 0 | 0 | 17 | 130 | 5 | 3 |
| **2** | 89 | 1,102 | 111 | 49 | 25 | 258 | 23 | 11 | 2 | 78 | 10 | 0 | 4 | 133 | 13 | 3 | 0 | 2 | 0 | 1 | 5 | 141 | 13 | 8 |
| **3** | 20 | 467 | 34 | 24 | 38 | 594 | 58 | 24 | 1 | 89 | 9 | 1 | 3 | 129 | 10 | 6 | 0 | 0 | 0 | 6 | 3 | 142 | 11 | 15 |
| **4** | 69 | 1,035 | 76 | 72 | 25 | 418 | 28 | 24 | 9 | 90 | 4 | 4 | 8 | 139 | 7 | 10 | 1 | 1 | 0 | 11 | 10 | 155 | 7 | 34 |
| **5** | 78 | 903 | 68 | 93 | 45 | 739 | 52 | 46 | 3 | 88 | 4 | 5 | 4 | 140 | 9 | 26 | 0 | 1 | 2 | 31 | 5 | 156 | 10 | 51 |
| **6** | 26 | 282 | 15 | 66 | 23 | 297 | 8 | 25 | 10 | 128 | 2 | 15 | 12 | 158 | 7 | 36 | 4 | 72 | 6 | 47 | 12 | 180 | 8 | 45 |
| **7** | 24 | 291 | 22 | 56 | 17 | 186 | 10 | 22 | 10 | 151 | 7 | 16 | 11 | 178 | 14 | 40 | 4 | 86 | 9 | 44 | 13 | 205 | 14 | 59 |
| **8** | 38 | 398 | 33 | 83 | 12 | 183 | 11 | 20 | 14 | 167 | 10 | 23 | 21 | 270 | 27 | 65 | 8 | 100 | 11 | 55 | 18 | 202 | 14 | 86 |
| **9** | 34 | 449 | 61 | 119 | 18 | 174 | 11 | 30 | 15 | 178 | 13 | 27 | 25 | 334 | 45 | 83 | 9 | 118 | 24 | 82 | 20 | 196 | 24 | 87 |
| **10** | 36 | 298 | 49 | 112 | 16 | 156 | 13 | 35 | 22 | 167 | 18 | 36 | 23 | 217 | 33 | 80 | 12 | 107 | 25 | 86 | 24 | 206 | 25 | 88 |
| **11** | 59 | 462 | 62 | 137 | 18 | 151 | 16 | 37 | 30 | 208 | 25 | 36 | 41 | 343 | 43 | 99 | 25 | 158 | 26 | 91 | 30 | 200 | 32 | 102 |
| **12** | 51 | 330 | 61 | 145 | 21 | 156 | 23 | 38 | 25 | 182 | 33 | 48 | 38 | 256 | 49 | 100 | 23 | 145 | 38 | 106 | 33 | 202 | 35 | 120 |
| **13** | 24 | 278 | 54 | 151 | 23 | 153 | 28 | 46 | 12 | 185 | 27 | 43 | 16 | 217 | 39 | 112 | 12 | 129 | 35 | 117 | 17 | 210 | 41 | 116 |
| **14** | 24 | 265 | 53 | 154 | 14 | 146 | 19 | 45 | 15 | 161 | 25 | 44 | 16 | 209 | 38 | 117 | 12 | 139 | 34 | 123 | 18 | 199 | 38 | 110 |
| **15** | 34 | 498 | 62 | 162 | 13 | 123 | 13 | 57 | 11 | 169 | 19 | 40 | 23 | 378 | 52 | 117 | 8 | 149 | 26 | 124 | 14 | 176 | 29 | 110 |
| **16** | 22 | 250 | 43 | 148 | 7 | 78 | 15 | 40 | 11 | 132 | 19 | 23 | 16 | 199 | 28 | 107 | 9 | 123 | 20 | 112 | 10 | 157 | 27 | 87 |
| **17** | 18 | 133 | 18 | 112 | 3 | 43 | 4 | 28 | 12 | 78 | 6 | 23 | 15 | 103 | 14 | 82 | 10 | 70 | 12 | 88 | 12 | 100 | 10 | 57 |
| **18+** | 6 | 74 | 6 | 72 | 0 | 1 | 0 | 9 | 4 | 37 | 1 | 18 | 5 | 54 | 5 | 55 | 4 | 44 | 4 | 64 | 3 | 46 | 3 | 0 |
| **Total** | 814 | 9,665 | 1,151 | 2,056 | 318 | 3,856 | 332 | 537 | 222 | 2,399 | 238 | 406 | 294 | 3,563 | 438 | 1,138 | 141 | 1,444 | 272 | 1,188 | 274 | 3,139 | 361 | 1,181 |

UW - underweight; NW- normal-weight; OW - overweight; OB - obese; IGF-1 - insulin-like growth factor-1; IGFBP-3 - IGF-binding protein 3; FSH - follicle-stimulating hormone; LH - luteinizing hormone; DHEA-S - dehydroepiandrosterone sulfate; SHBG - sex hormone-binding globulin; HOMA-IR - homeostatic model assessment for insulin resistance; TSH - thyroid-stimulating hormone; FT4 - free thyroxin.

**If. Multiple observations**

47·7% of the 8,629 children had multiple anthropometric observations. A detailed overview of the numbers of children with the number of observations is shown in Table S3. This table indicates the number of multiple observations the children underwent during the study period. 4,516 children only had a single observation, 405 children had two observations, 259 children had 3 observations etc. The maximum amount of observations was 17. The cumulative sample sizes are presented calculated downwards and upwards.

**Table S3. Number of longitudinal data available for the children**

| **Number of observations** | **Number of children** | **Cumulative sample size** | |
| --- | --- | --- | --- |
| 1 | 4,516 |  |  |
| 2 | 405 | 405 | 4,113 |
| 3 | 259 | 664 | 3,708 |
| 4 | 256 | 920 | 3,449 |
| 5 | 195 | 1,115 | 3,193 |
| 6 | 252 | 1,367 | 2,998 |
| 7 | 336 | 1,703 | 2,746 |
| 8 | 509 | 2,212 | 2,410 |
| 9 | 444 | 2,656 | 1,901 |
| 10 | 309 | 2,965 | 1,457 |
| 11 | 327 | 3,292 | 1,148 |
| 12 | 316 | 3,608 | 821 |
| 13 | 234 | 3,842 | 505 |
| 14 | 173 | 4,015 | 271 |
| 15 | 54 | 4,069 | 98 |
| 16 | 42 | 4,111 | 44 |
| 17 | 2 | 4,113 | 2 |

**II. The CrescNet registry**

**IIa. Description of the registry**

CrescNet is a German pediatric registry[^7^](#_ENREF_7) with nationwide 435 contributing pediatric practices and 31 contributing hospitals or ambulances. Data are transferred from 153 primary care pediatricians or institutions from Germany[^8^](#_ENREF_8). The patient registry encompasses data of 933,324 participants (479,700 boys, 453,624 girls) with in total 4,999,225 observations (2,570,863 boys, 2,428,362 girls). The children are asked for participation without any selection when seen by in practices. Data were collected from January 1999 to February 2020. Data of more than 5% of all children in Germany are represented in the CrescNet registry starting from the year 2000.

Anthropometric measurements of children are performed during pediatric consultations such as well-child visits, medical checkups as well as consultations for acute illnesses by medical assistance personal who were trained on the measuring methods and have been provided with a manual[^8^](#_ENREF_8). Height is assessed with uniform scales (“Dr. Keller series”) measuring to the closest of 0·1 cm. Weight was assessed of children wearing light underwear using calibrated digital scales with an accuracy of +/-100 g. In order to control for plausibility of the data, height, weight or body mass index (BMI) standard deviation score (SDS) outside ±5 SDS or a change higher than ±1 SDS per year of those parameters or a >3cm decline in height were rejected.

The guardians/parents were informed about the registry and asked for consent by their pediatricians. Data were documented in the local practice and transferred automatically to the CrescNet registry in a pseudonymized manner. Participants and parents can withdraw their consent at any time point leading to complete deletion of their data. The registry was approved by the Federal Saxonian Data Protection Authority and is registered at the clinical trials database (NCT03072537).

**IIb. Inclusion criteria for the generation of height reference values for children with obesity**

The inclusion criteria are adopted from the study described above (Additional file 1, Ic). In Figure S3 the inclusion criteria for CrescNet data for the generation of height reference values for children with obesity are presented step-wise in a flow chart. Observations from children aged 0–20 years (maximum one observation per year) with a height standard deviation score (SDS) from -2·5 to 4·0 and a body mass index (BMI) SDS higher than -3·5 and recorded age of gestation were initially included. Children suffering from diseases such as type 1 diabetes (T1DM), syndromes and other conditions with severe disability (permanent immobility, microcephaly etc.) and children with manifested precocious puberty were excluded, as well as individuals taking medications affecting growth (e.g. growth hormone, systemic glucocorticoids, immunosuppressives). Valproate medication was not recorded in the CrescNet registry. Data from prematurely born babies (week of gestation<38) below the age of 2 years were excluded as by then they are supposed to have caught up in height[^6^](#_ENREF_6). From those observations only one observation was randomly selected per individual and children were classified into normal-weight (BMI SDS from -1·28 to 1·28) and obese (BMI SDS >1·88). In total 12,703 observations 6,108 from boys (**♂**) and 6,595 girls (**♀**) were used for the generation of reference values for children with obesity.


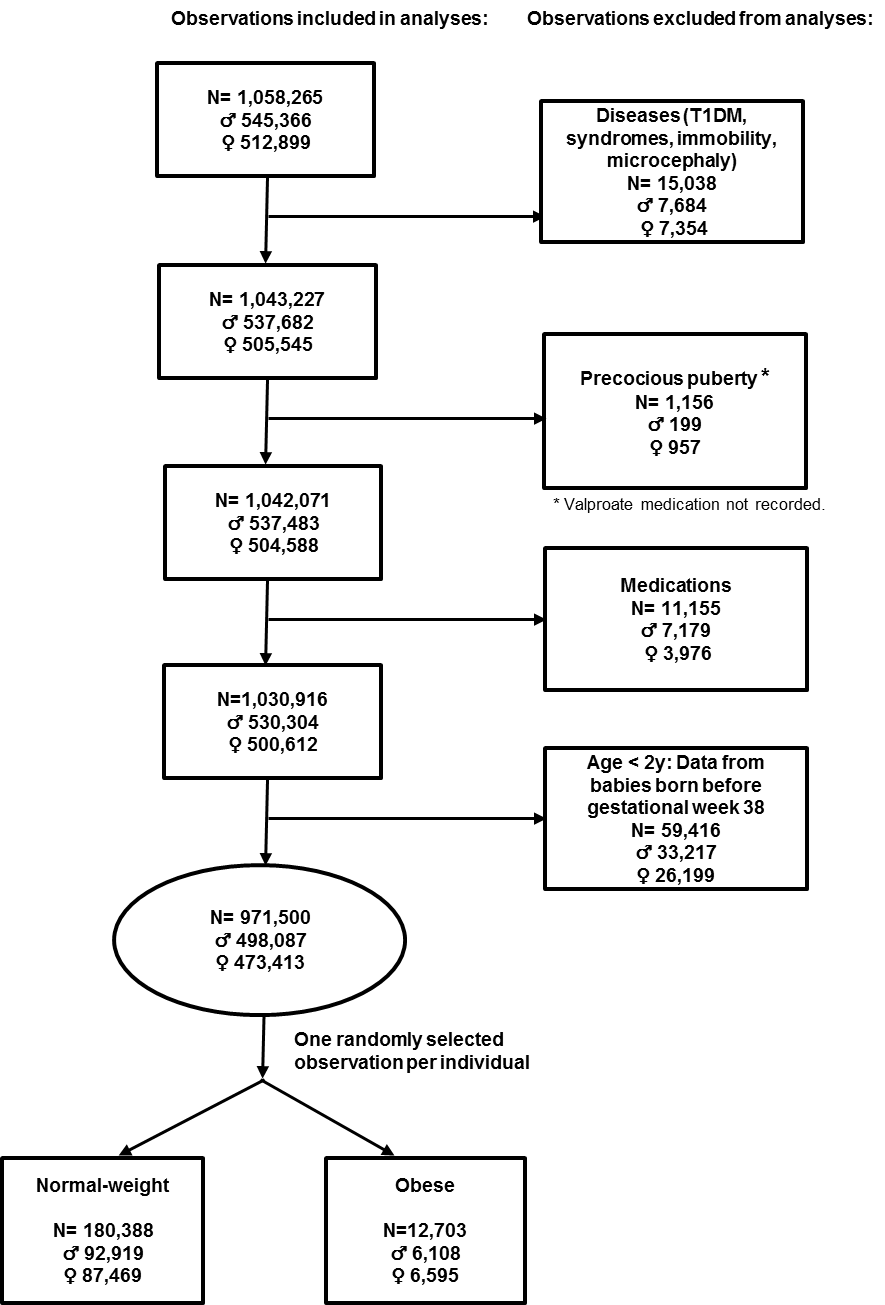


**Figure S3. Inclusion criteria for CrescNet data for the generation of height reference values for children with obesity**

Observations from children aged 0–20 years with a height standard deviation score (SDS) from -2·5 to 4·0 and a body mass index SDS higher than -3·5 were initially included. Then observations were excluded due to diseases, conditions of precocious puberty, medication or premature birth (until the age of 2 years). Here N indicates the number of observations, also separately presented for boys (**♂**) and for girls (**♀**). Finally, only one randomly selected observation of each child was used for the generation of reference values. Children were classified into normal-weight and obese. Data from 6,108 boys and 6,595 girls with obesity were used for the generation of height reference values.

**III. Pubertal stages of children of different weight categories**

Pubertal stages were evaluated according to pubic hair and testicular volume or breast stage the Tanner criteria[^9^](#_ENREF_9)^,^[^10^](#_ENREF_10) as shown in Table S4. The mean ages of children of different weight categories in the different pubertal stages 1-5 were analyzed. Results are shown in Table S5. Girls with obesity entered puberty 9·1 months and menarche 8·9 months earlier than normal-weight girls. Boys with obesity entered puberty 6·6 months later.

**Table S4. Evaluation of pubertal stage according to Tanner criteria**

|  | **Boys** | | | **Girls** | | |
| --- | --- | --- | --- | --- | --- | --- |
| **Pubertal stage** | **Pubic hair** | **TV (cm³)** |  | **Pubic hair** | **Breast** |  |
| **1** | PH=1 | TV≤3 |  | PH=1 | B=1 |  |
| **2** | PH≤2 | 4≤TV≤10 | or | PH≤3 | B=2 | or |
|  | PH≥2 | TV≤3 |  | PH≥2 | B=1 |  |
| **3** | PH=3 | TV≥4 |  | 2≤PH≤4 | B=3 |  |
| **4** | PH=4 | TV≥4 |  | PH≥3 | B=4 |  |
| **5** | PH≥5 | TV≥7 |  | PH≥4 | B=5 |  |

PH – pubic hair; TV – testicular volume; B – breast stage

**Table S5. Mean age of pubertal stages and onset of menarche in children with underweight, normal-weight, overweight or obesity older than 5 years** Data are presented as mean age in years (y) with standard error of the mean (SEM) and the number of subjects (N). For each pubertal stage mean ages of normal-weight children were compared with mean ages of children with underweight, overweight or obesity using multiple unpaired t-tests (two-sided) with Holm-Šídák correction for multiple comparison. Unadjusted *P*-values are shown. *P*-values are highlighted in bold when remaining <0·05 after adjustment for multiple testing.

|  |  | **UW** | | **NW** | | **OW** | | **OB** | | **NW vs. UW** | **NW vs. OW** | **NW vs. OB** |
| --- | --- | --- | --- | --- | --- | --- | --- | --- | --- | --- | --- | --- |
|  | **Pubertal stage/ menarche** | **Age, y (SEM)** | **N** | **Age, y (SEM)** | **N** | **Age, y (SEM)** | **N** | **Age, y (SEM)** | **N** | ***P*-value** | ***P*-value** | ***P*-value** |
|  | P1 | 8·75 (0·2) | 129 | 8·36 (0·06) | 1133 | 9·55 (0·19) | 112 | 8·96 (0·11) | 294 | **< 0·0001** | **< 0·0001** | **< 0·0001** |
|  | P2 | 11·82 (0·3) | 43 | 11·03 (0·1) | 320 | 11·33 (0·17) | 85 | 11·58 (0·12) | 187 | **< 0·0001** | **0.00070** | **< 0·0001** |
| **Boys** | P3 | 14·16 (0·52) | 7 | 13·22 (0·15) | 86 | 13·12 (0·39) | 21 | 13·16 (0·14) | 83 | **< 0·0001** | 0.63 | **0.0079** |
|  | P4 | 15·58 (0·35) | 13 | 14·77 (0·1) | 140 | 14·46 (0·24) | 19 | 14·35 (0·15) | 74 | **< 0·0001** | **0.00053** | **< 0·0001** |
|  | P5 | 15·59 (0·16) | 20 | 15·48 (0·05) | 300 | 15·23 (0·17) | 36 | 15·33 (0·13) | 109 | 0.0075 | **< 0·0001** | **< 0·0001** |
|  | P1 | 8·51 (0·20) | 100 | 7·64 (0·06) | 952 | 8·42 (0·18) | 80 | 7·82 (0·11) | 233 | **< 0·0001** | **< 0·0001** | 0.094 |
|  | P2 | 11·53 (0·14) | 50 | 10·71 (0·07) | 344 | 10·04 (0·15) | 81 | 9·95 (0·14) | 160 | **< 0·0001** | **< 0·0001** | **< 0·0001** |
| **Girls** | P3 | 12·54 (0·39) | 10 | 12·14 (0·09) | 195 | 11·53 (0·18) | 47 | 11·76 (0·13) | 91 | 0.3368 | **0.0039** | **0.0069** |
|  | P4 | 15·02 (0·23) | 19 | 13·96 (0·13) | 188 | 12·92 (0·30) | 26 | 13·59 (0·14) | 100 | **0.0098** | **0.0039** | 0.031 |
|  | P5 | 15·75 (0·19) | 30 | 15·31 (0·05) | 492 | 15·13 (0·13) | 80 | 15·10 (0·09) | 307 | **0.0241** | 0.17 | **0.00046** |
|  | menarche | 13·13 (0·22) | 39 | 12·55 (0·05) | 466 | 12·20 (0·15) | 71 | 11·81 (0·07) | 310 | **0.0037** | 0.023 | **< 0·0001** |

UW - underweight; NW- normal-weight; OW - overweight; OB – obese; vs. – versus; y –years; SEM - standard error of the mean; N – sample size, P – Pubertal stage. **IV. Parental data and birth parameters**

**IVa. Parental height**

Parental height and body mass index (BMI) has been assessed by self-reports.

Height SDS adjusted to mid-parental height SDS (Height_parental adj_ SDS):

First, mid-parental height SDS was calculated as mean parental height +6·5 cm for boys, and -6·5 cm for girls, with subsequent standardization to SDS using the reference data for 18-years olds^[11](#_ENREF_11)^. Height SDS adjusted to mid-parental height SDS (Height_parental adj_ SDS) was calculated as the difference of the child’s height SDS and the mid-parental height SDS. Values between -2·5 and 4·0 were included. Additionally, heights of the parents have been investigated separately. The fathers of children with obesity were approximately 2 cm shorter, the mothers approximately 1 cm (Figure S4).

**Figure S4. Comparison of parental height in children with normal-weight or obesity**

Parental height of children was compared between children with normal-weight (NW) and obesity (OB). Only the first observation of a child was used. The weight status of the father and the mother was categorized according to the body mass index (BMI) in kg/m² in BMI<25 and BMI≥25. a: Paternal height; b: Maternal height. Data are shown as mean with standard error. *P*-values corrected for multiple comparison are given for significant differences (p<0·05) as assessed by two-way ANOVA and Fishers Least Significant Difference test combined with a Holm-Šídák multiple comparison test.

**IVb. Birth parameters**

Analyses in children regardless of the weight showed that higher birth length as well as higher birth weight is associated with increased height in later childhood between age 4-14·999 years (Figure S5). Children were classified according to birth length standard deviation scores (SDS) and birth weight SDS, into born small for gestational age (SGA; ≤10^th^ percentiles of the cohort), appropriate for gestational age (AGA; <90^th^ and >10^th^ percentile) and large for gestational age (LGA; ≥90^th^ percentiles)[^12^](#_ENREF_12). SDS for birth length and birth weight were calculated according to Voigt and corrected for gestational age[^13^](#_ENREF_13). Data of children born before 38^th^ week of gestation were excluded from the analyses.

Due to the observed associations with height we asked if our children with obesity, who present increased height, were already taller or heavier at birth. In particular girls with obesity in ages 4-14·99 years, were heavier and taller at birth (Table S6). Their parents had a higher BMI and were slightly smaller than the parents of normal-weight children.

**Figure S5. The impact of birth length and birth weight on later height standard deviation scores (SDS)**

Newborns were categorized into born small for gestational age (SGA), appropriate for gestational age (AGA) and large for gestational age (LGA) according to the body length and body weight[^12^](#_ENREF_12). Height SDS of boys (a) and girls (b) between the ages 4-14·99 years are shown as mean with standard error. *P*-values corrected for multiple comparison are given for significant differences (p<0·05) as assessed by two-way ANOVA and Fishers Least Significant Difference test combined with a Holm-Šídák multiple comparison test.

**Table S6. Comparison of birth-related and parental parameters between children with normal-weight and obesity of ages 4-14 years** Birth-related and parental parameters of children, who were obese (OB) or normal-weight (NW) between ages of 4-14·99 years, were compared using unpaired t-test (two-sided) with Holm-Šídák correction for multiple comparison. Unadjusted *P*-values <0·05 are shown. *P*-values are highlighted in bold when remaining <0·05 after adjustment for multiple testing.

|  | **NW children** | | **OB children** | |  |
| --- | --- | --- | --- | --- | --- |
| **Boys (4-14 years)** | **Mean (SEM)** | **N** | **Mean (SEM)** | **N** | ***P-*value** |
| **Birth weight, g** | 3579·06 (14·26) | 1,034 | 3627·68 (23·45) | 494 | 0·064 |
| **Birth weight SDS** | 0·03 (0·03) | 1,034 | 0·09 (0·05) | 494 | 0·36 |
| **Birth length, cm** | 51·11 (0·07) | 1,024 | 51·36 (0·11) | 489 | 0·039 |
| **Birth length SDS** | -0·50 (0·03) | 1,024 | -0·44 (0·04) | 489 | 0·23 |
| **Paternal BMI, kg/m²** | 25·94 (0·15) | 578 | 29·42 (0·36) | 271 | **<0·0001** |
| **Maternal BMI, kg/m²** | 24·15 (0·18) | 654 | 29·91 (0·4) | 304 | **<0·0001** |
| **Paternal height, cm** | 180·56 (0·29) | 636 | 178·84 (0·42) | 345 | **0·00059** |
| **Maternal height, cm** | 167·90 (0·25) | 673 | 166·28 (0·34) | 366 | **0·00012** |
|  | **NW children** |  | **OB children** |  |  |
| **Girls (4-14 years)** | **Mean (SEM)** | **N** | **Mean (SEM)** | **N** | ***P-*value** |
| **Birth weight, g** | 3434·72 (14·24) | 968 | 3547·54 (25·45) | 480 | **<0·0001** |
| **Birth weight SDS** | 0·02 (0·03) | 968 | 0·27 (0·06) | 480 | **<0·0001** |
| **Birth length, cm** | 50·11 (0·07) | 962 | 50·62 (0·12) | 479 | **<0·0001** |
| **Birth length SDS** | -0·62 (0·03) | 962 | -0·41 (0·05) | 479 | **0·00021** |
| **Paternal BMI, kg/m²** | 26·06 (0·16) | 539 | 29·31 (0·36) | 235 | **<0·0001** |
| **Maternal BMI, kg/m²** | 24·24 (0·18) | 626 | 30·71 (0·44) | 297 | **<0·0001** |
| **Paternal height, cm** | 180·1 (0·29) | 605 | 177·96 (0·46) | 310 | **<0·0001** |
| **Maternal height, cm** | 167·71 (0·26) | 642 | 166·46 (0·37) | 354 | **0·0047** |

SEM - standard error of the mean; N - number of individuals; SDS - standard deviation score; BMI - body mass index

**IVc. Obesity-related increase in height in early childhood is independent from parental height or birth parameters**


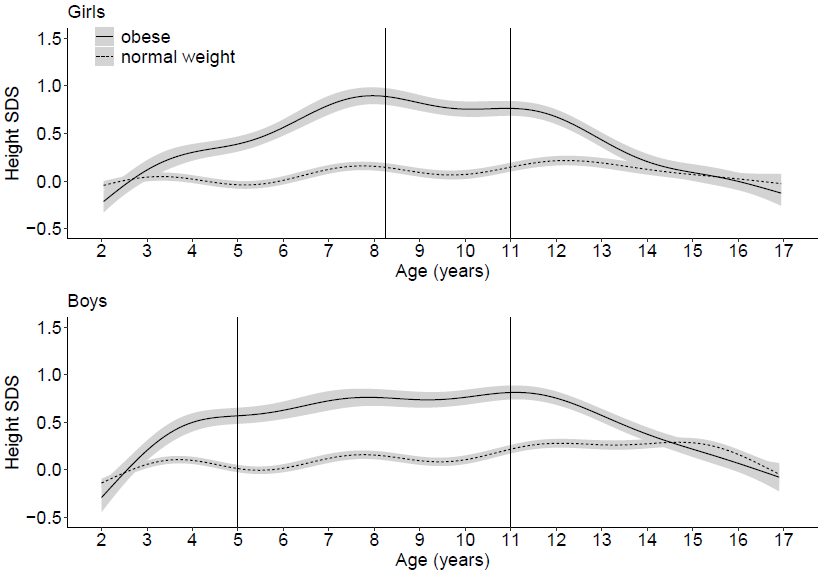


**Figure S6. Trends of height SDS between normal-weight and children with obesity** Trends of height SDS between normal-weight children and children with obesity were modeled using generalized additive mixed models. The optimal age-dependent cut-offs were defined visually at the age of 5 and 11 years of age in boys and 8·25 and 11 years of age in girls.

**Table S7·: Univariate and multivariate regression analyses in three different age intervals in boys and in girls** The effect of mid-parental height SDS and birth length SDS on height SDS was analysed.

|  |  | **Boys** |  |  |  |  |  | **Girls** |  |  |  |  |  |
| --- | --- | --- | --- | --- | --- | --- | --- | --- | --- | --- | --- | --- | --- |
| **Age Interval 1** | | **2·0-4·99 years** | | |  |  |  | **2·0-8·249 years** | | |  |  |  |
|  |  | **Normal-weight** | | | **Obese (Δ to Normal-weight)** | | | **Normal-weight** | | | **Obese (Δ to Normal-weight)** | | |
| **Regression** |  | **N** | **β** | ***p*** | **N** | **β** | ***p*** | **N** | **β** | ***p*** | **N** | **β** | ***p*** |
| Univariate | Age vs. Height SDS | 828 (1607) | 0·09 | < 0·0001 | 58 (85) | 0·35 | < 0·0001 | 884 (3132) | 0·03 | < 0·0001 | 146 (299) | 0·14 | < 0·0001 |
| Multivariate | Age vs. Height SDS | 828 (1607) | 0·10 | < 0·0001 | 58 (85) | 0·35 | < 0·0001 | 884 (3132) | 0·03 | < 0·0001 | 146 (299) | 0·15 | < 0·0001 |
|  |  |  |  | **Normal-weight and Obese** | |  |  |  | **Normal-weight and Obese** | | |  |  |
|  | **Covariates** |  |  | **β** | ***p*** |  |  |  |  | **β** | ***p*** |  |  |
|  | Mid-parental height SDS |  |  | 0·46 | < 0·0001 |  |  |  |  | 0·47 | < 0·0001 |  |  |
|  | Birth length SDS |  |  | 0·25 | < 0·0001 |  |  |  |  | 0·24 | < 0·0001 |  |  |
| **Age Interval 2** | | **5·0-10·99 years** | | | |  |  | **8·25-10·99 years** | | | |  |  |
|  |  | **Normal-weight** | | | **Obese (Δ to Normal-weight)** | | | **Normal-weight** | | | **Obese (Δ to Normal-weight)** | | |
| **Regression** |  | **N** | **β** | ***p*** | **N** | **β** | ***p*** | **N** | **β** | ***p*** | **N** | **β** | ***p*** |
| Univariate | Age vs. Height SDS | 678 (1791) | 0·04 | < 0·0001 | 208 (319) | 0·01 | 0·23 | 296 (540) | 0·04 | 0·003 | 133 (171) | -0·06 | 0·09 |
| Multivariate | Age vs. Height SDS | 678 (1791) | 0·04 | < 0·0001 | 208 (319) | 0·01 | 0·31 | 296 (540) | 0·04 | 0·002 | 133 (171) | -0·06 | 0·09 |
|  |  |  |  | **Normal-weight and Obese** | |  |  |  | **Normal-weight and Obese** | | |  |  |
|  | **Covariates** |  |  | **β** | ***p*** |  |  |  |  | **β** | ***p*** |  |  |
|  | Mid-parental height SDS |  |  | 0·48 | < 0·0001 |  |  |  |  | 0·48 | < 0·0001 |  |  |
|  | Birth length SDS |  |  | 0·19 | 0·0004 |  |  |  |  | 0·19 | 0·0004 |  |  |
| **Age Interval 3** | | **11·0-17·0 years** | | |  |  |  | **11·0-17·0 years** | | |  |  |  |
|  |  | **Normal-weight** | | | **Obese (Δ to Normal-weight)** | | | **Normal-weight** | | | **Obese (Δ to Normal-weight)** | | |
| **Regression** |  | **N** | **β** | ***p*** | **N** | **β** | ***p*** | **N** | **β** | ***p*** | **N** | **β** | ***p*** |
| Univariate | Age vs. Height SDS | 365 (887) | -0·01 | 0·20 | 263 (385) | -0·2 | < 0·0001 | 366 (873) | -0·04 | < 0·0001 | 265 (420) | -0·09 | < 0·0001 |
| Multivariate | Age vs. Height SDS | 365 (887) | -0·01 | 0·29 | 263 (385) | -0·1 | < 0·0001 | 366 (873) | -0·04 | < 0·0001 | 265 (420) | -0·09 | < 0·0001 |
|  |  |  |  | **Normal-weight and Obese** | |  |  |  | **Normal-weight and Obese** | | |  |  |
|  | **Covariates** |  |  | **β** | ***p*** |  |  |  |  | **β** | ***p*** |  |  |
|  | Mid-parental height SDS |  |  | 0·59 | < 0·0001 |  |  |  |  | 0·53 | < 0·0001 |  |  |
|  | Birth length SDS |  |  | 0·16 | < 0·0001 |  |  |  |  | 0·24 | < 0·0001 |  |  |

N - number of individuals with the number of observations in brackets, SDS - standard deviation score; Δ to Normal weight - difference compared to normal-weight children; *p* - *p*-value; β – unstandardized beta

**V. Non-parametric modelling of growth patterns and IGF-1**

We analyzed the age trend for height standard deviation scores (SDS), growth velocity and insulin-like growth factor-1 (IGF-1) non-parametrically by applying generalized additive models taking advantage of longitudinal multiple measurements per subject by adding a random effect for the subjects assuming a simple Gaussian random effect.

These models (Figure S7) provide the same growth and IGF-1 patterns as presented in Figure 1-3: Height SDS and growth velocity are increased in children with obesity before puberty. During puberty, growth speed is reduced in the obesity group and height SDS declines consequently. Also, the increase in growth velocity during mid-pubertal age was blunted compared to the normal-weight group.

IGF-1 (ng/mL)

**Figure S7. Non-parametric modelling of growth patterns** Modelling of growth patterns in the weight groups over age taking advantage of the longitudinal data and at the same time not introducing bias due to unweighted effects of overrepresented subjects with repetitive measurements and to incorporate adjustments. These models do confirm a considerable higher height SDS during early childhood, both for boys and girls with obesity (red) with subsequent catch-down and regression to the mean of the normal-weight peers (blue) (a). Similarly, in the obese group growth velocity is accelerated in early childhood, but does not increase during pubertal age and decreases earlier compared to the normal-weight group (b). Also, IGF-1 levels showed a similar pattern with higher levels in early childhood and lower levels during puberty in the obese group (c).

**VI. Serum levels of additional hormones in children with normal-weight and obesity in relation to growth velocities**

**Figure S8. Serum levels of sex hormones in children with normal-weight and obesity in relation to growth velocities**

Serum sex hormone levels of children with obesity (OB, full black squares) and normal-weight (NW, open grey circles) are presented. The dotted- and dashed-lined curves present the growth velocities (GV) in cm per year for individuals with normal-weight and obesity, respectively, from whom sex steroid measurements were available. Sample sizes are provided in Additional file 1, Ie. a, b: Serum luteinizing hormone (LH); c, d: Serum follicle-stimulating hormone (FSH); e, f: Serum sex hormone-binding globulin (SHBG); g, h: Serum dehydroepiandrosterone sulfate (DHEA-S). Data are shown as mean with standard error; Asterisks (*) mark significant (p<0·05) differences between NW and OB children assessed by multiple t-tests combined with a Holm-Šídák multiple comparison test.

**Figure S9. Serum levels of thyroid hormones in children with normal-weight and obesity in relation to growth velocities**

Serum thyroid hormone levels of children with obesity (OB, full black squares) and normal-weight (NW, open grey circles) are presented. The dotted- and dashed-lined curves present the growth velocities (GV) in cm per year for individuals with normal-weight and obesity, respectively, from whom thyroid hormone measurements were available. Data are shown as mean with standard error; Asterisks (*) mark significant (p<0·05) differences between NW and OB children assessed by multiple t-tests combined with a Holm-Šídák multiple comparison test. Sample sizes are provided in Additional file 1, Ie. a, b: Serum thyroid-stimulating hormone (TSH); c, d: Serum free thyroxin (FT4).

**VII. Height, growth and endocrine factors in underweight and overweight children**

The group of underweight children had lower mean height (SDS) before puberty, followed by catch-up during ages 14-16 years and finally reaching similar heights as the other weight groups (Figure S10). This delayed pubertal growth spurt was even more obvious to discern by growth velocities (Figure S11) and was accompanied by lower levels of IGF-1 (Figure S12). Sex steroid, but not FSH levels appeared also slightly lower than in normal-weight children (Figure S13-15). The SHBG levels, however, were higher in the underweight group and overall showed the clearest dependence on weight group.

**Figure S10. Height parameters of children with underweight, normal-weight, overweight or obesity**

Parameters of height for children with underweight (UW, grey empty triangles), normal-weight (NW, empty grey circles), overweight (OW, dark grey empty squares) or obesity (OB, full black squares) in the ages from 0-18+ are shown. Sample sizes are provided in Additional file 1, Ie. a, b: Total height; c, d: Height standard deviation score (SDS); e, f: Height SDS relative to mid-parental height SDS (Height_parental adj_ SDS). Data are shown as mean with standard error.

**Figure S11. Growth velocities of children with underweight, normal-weight, overweight or obesity**

Growth velocities for children with underweight (UW, grey empty triangles), normal-weight (NW, empty grey circles), overweight (OW, dark grey empty squares) or obesity (OB, full black squares) are presented. Sample sizes are provided in Additional file 1, Ie. a, b: Growth velocities in cm per year between the ages of 2-18+ years; c, d: Growth velocity standard deviation score (SDS) according to Reinken^[14](#_ENREF_14)^. Data are shown as mean with standard error.

**Figure S12. Serum IGF-1 and metabolic parameters of children with underweight, normal-weight, overweight or obesity**

Serum hormone levels of children with underweight (UW, bright grey open triangle), normal-weight (NW, grey open circles), overweight (OW, dark grey open squares) or obesity (OB, black full squares) aged 5-18+ years are presented. Sample sizes are provided in Additional file 1, Ie. a, b: Serum insulin-like growth factor-1 (IGF-1); c, d: Fasting serum insulin; e, f: Homeostatic Model Assessment for Insulin Resistance (HOMA-IR). Data are shown as mean with standard error.

**Figure S13. Serum sex hormone levels of children with underweight, normal-weight, overweight or obesity (I)**

Serum hormone levels of children with underweight (UW, bright grey open triangle), normal-weight (NW, grey open circles), overweight (OW, dark grey open squares) or obesity (OB, black full squares) aged 5-18+ years are presented. Sample sizes are provided in Additional file 1, Ie. a, b: Serum testosterone; c, d: Serum estradiol. Data are shown as mean with standard error.

**Figure S14. Serum sex hormone levels of children with underweight, normal-weight, overweight or obesity (II)**

Serum sex hormone levels of children with underweight (UW, bright grey open triangle), normal-weight (NW, grey open circles), overweight (OW, dark grey open squares) or obesity (OB, black full squares) aged 5-18+ years are presented. Sample sizes are provided in Additional file 1, Ie. a, b: Serum luteinizing hormone (LH); c, d: Serum follicle-stimulating hormone (FSH); e, f: Serum sex hormone-binding globulin (SHBG); g, h: Serum dehydroepiandrosterone sulfate (DHEA-S). Data are shown as mean with standard error.

**Figure S15. Serum thyroid hormone levels of children with underweight, normal-weight, overweight or obesity**

Serum thyroid hormone levels of children with underweight (UW, bright grey open triangle), normal-weight (NW, grey open circles), overweight (OW, dark grey open squares) or obesity (OB, black full squares) aged 5-18+ years are presented. Sample sizes are provided in Additional file 1, Ie. a, b: Serum thyroid-stimulating hormone (TSH); c, d: Serum free thyroxine (FT4). Data are shown as mean with standard error.

**VIII. Online tools: Height reference values for children with obesity**


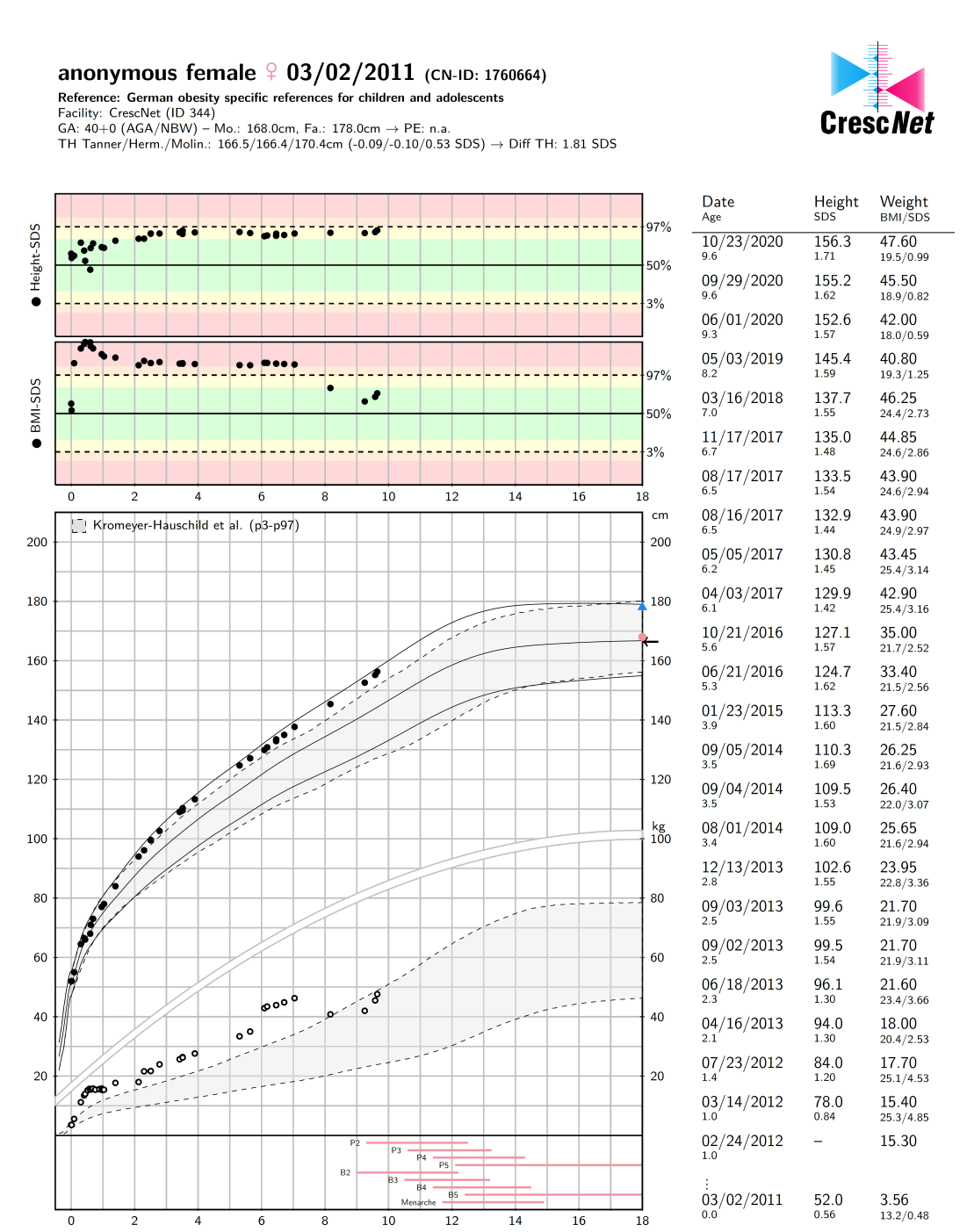


**Figure S16. Calculating height SDS for a child with obesity at the CrescNet website using the novel reference values**

This figure exemplarily shows an output file generated at the CrescNet website[^15^](#_ENREF_15) using the novel height reference values for children with obesity. In the left upper graph the height standard deviation scores (SDS) of the child according to reference values for children with obesity and the body mass index (BMI) SDS of the child according to Kromeyer-Hauschild^[11](#_ENREF_11)^ are visualized. The lower graph shows the total height of the child at several ages (full circles) compared to the novel height percentiles (3^rd^, 50^th^ and 97^th^ percentile) for obesity (solid lines) and compared to the 3^rd^ and the 97^th^ percentiles according Kromeyer-Hauschild^[11](#_ENREF_11)^ (dashed lines, highlighted in grey). In the lower part of this graph the weight of the child (open circles) compared to the weight percentiles (3^rd^ and 97^th^ percentile) (dashed lines, highlighted in grey) according Kromeyer-Hauschild^[11](#_ENREF_11)^ is depicted. On the right side the height, weight and BMI data from the child at the different ages are given, including the height SDS according to the novel height reference values for children with obesity and the BMI SDS values according to Kromeyer-Hauschild^[11](#_ENREF_11)^.


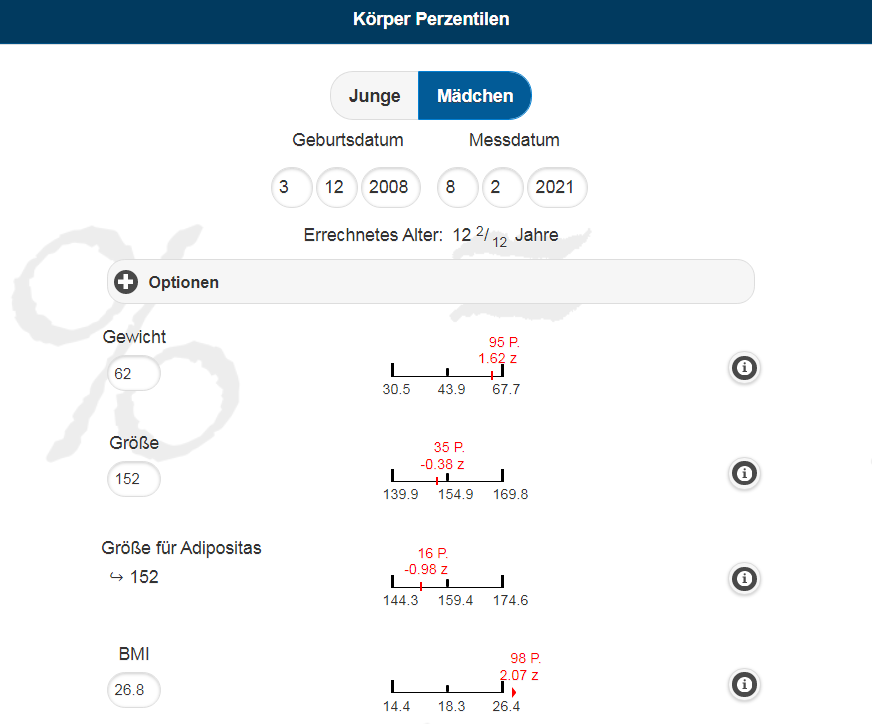


**Figure S17. Calculation of height percentiles for a child with obesity using the Ped(Z) Pediatric Calculator application**

This screenshot (in German) exemplarily shows a calculation of height percentiles using the Ped(Z) application available at the Pediatric Calculator website[^16^](#_ENREF_16). If a child suffers from obesity (body mass index (BMI) standard deviation score (z) >1·88), the novel height percentiles (P) together with the z-score for children with obesity automatically appear below the height percentiles according to Kromeyer-Hauschild[^11^](#_ENREF_11).

**Additional file 1 References**

[1]. Poulain T, Baber R, Vogel M, et al. The LIFE Child study: a population-based perinatal and pediatric cohort in Germany. *Eur J Epidemiol* 2017; **32**(2): 145-58.

[2]. Quante M, Hesse M, Dohnert M, et al. The LIFE child study: a life course approach to disease and health. *BMC Public Health* 2012; **12**: 1021.

[3]. Landgraf K, Rockstroh D, Wagner IV, et al. Evidence of early alterations in adipose tissue biology and function and its association with obesity-related inflammation and insulin resistance in children. *Diabetes* 2015; **64**(4): 1249-61.

[4]. Landgraf K, Friebe D, Ullrich T, et al. Chemerin as a mediator between obesity and vascular inflammation in children. *J Clin Endocrinol Metab* 2012; **97**(4): E556-64.

[5]. Landgraf K, Klöting N, Gericke M, et al. The obesity-susceptibility gene TMEM18 promotes adipogenesis through activation of PPARG. *Cell Rep* 2020; **33**(3).

[6]. Euser AM, de Wit CC, Finken MJJ, Rijken M, Wit JM. Growth of preterm born children. *Horm Res Paediatr* 2008; **70**(6): 319-28.

[7]. Keller E, Gausche R, Meigen C, Keller A, Burmeister J, Kiess W. Auxological computer based network for early detection of disorders of growth and weight attainment. *J Pediatr Endocrinol Metab* 2002; **15**(2): 149-56.

[8]. Geserick M, Vogel M, Gausche R, et al. Acceleration of BMI in Early Childhood and Risk of Sustained Obesity. *N Engl J Med* 2018; **379**(14): 1303-12.

[9]. Marshall WA, Tanner JM. Variations in pattern of pubertal changes in girls. *Arch Dis Child* 1969; **44**(235): 291-303.

[10]. Marshall WA, Tanner JM. Variations in the pattern of pubertal changes in boys. *Arch Dis Child* 1970; **45**(239): 13-23.

[11]. Kromeyer-Hauschild K, Wabitsch M, Kunze D, et al. Percentiles of body mass index in children and adolescents evaluated from different regional German studies. *Monatsschrift Kinderheilkunde* 2001; **149**(8): 807-18.

[12]. Voigt M, Rochow N, Jahrig K, Straube S, Hufnagel S, Jorch G. Dependence of neonatal small and large for gestational age rates on maternal height and weight--an analysis of the German Perinatal Survey. *J Perinat Med* 2010; **38**(4): 425-30.

[13]. Voigt M, Fusch C, Olbertz D, et al. Analyse des Neugeborenenkollektivs der Bundesrepublik Deutschland. *Geburtshilfe Frauenheilkd* 2006; **66**(10): 956-70.

[14]. Reinken L, van Oost G. Longitudinale Körperentwicklung gesunder Kinder von 0 bis 18 Jahren. *Klin Padiatr* 1992; **204**(03): 129-33.

[15]. Beger C. CrescNet. https://crescnet.medizin.uni-leipzig.de (accessed June 2021).

[16]. Gräfe D. Ped(Z) Pediatric Calculator. https://bmi.pedz.de (accessed June 2021).
